# Localized fluorescent imaging of multiple proteins on individual extracellular vesicles using rolling circle amplification for cancer diagnosis

**DOI:** 10.1002/jev2.12025

**Published:** 2020-11-11

**Authors:** Junli Zhang, Jinjin Shi, Hongling Zhang, Yifan Zhu, Wei Liu, Kaixiang Zhang, Zhenzhong Zhang

**Affiliations:** ^1^ Henan Key Laboratory of Targeting Therapy and Diagnosis for Critical Diseases School of Pharmaceutical Sciences Zhengzhou University Zhengzhou 450001 China

**Keywords:** cancer diagnosis, cancer subtype differentiation, individual extracellular vesicles heterogeneity, localized fluorescent imaging, rolling circle amplification

## Abstract

Extracellular vesicles (EV) have attracted increasing attention as tumour biomarkers due to their unique biological property. However, conventional methods for EV analysis are mainly based on bulk measurements, which masks the EV‐to‐EV heterogeneity in tumour diagnosis and classification. Herein, a localized fluorescent imaging method (termed Digital Profiling of Proteins on Individual EV, DPPIE) was developed for analysis of multiple proteins on individual EV. In this assay, an anti‐CD9 antibody engineered biochip was used to capture EV from clinical plasma sample. Then the captured EV was specifically recognized by multiple DNA aptamers (CD63/EpCAM/MUC1), followed by rolling circle amplification to generate localized fluorescent signals. By‐analyzing the heterogeneity of individual EV, we found that the high‐dimensional data collected from each individual EV would provide more precise information than bulk measurement (ELISA) and the percent of CD63/EpCAM/MUC1‐triple‐positive EV in breast cancer patients was significantly higher than that of healthy donors, and this method can achieve an overall accuracy of 91%. Moreover, using DPPIE, we are able to distinguish the EV between lung adenocarcinoma and lung squamous carcinoma patients. This individual EV heterogeneity analysis strategy provides a new way for digging more information on EV to achieve multi‐cancer diagnosis and classification.

## 1 INTRODUCTION

Cancer diagnosis, classification and prognosis monitoring are keys for reducing cancer deaths. Current gold standard for clinical tumour diagnosis is mainly based on combination of imageological and histopathological examinations (Turkbey et al., 2011; Ueno et al., 2016). Despite their accuracy and general applicability for multi‐tumour diagnosis, they are usually invasive, complex, not sensitive enough for early‐stage cancer, and may increase risk of tumour metastasis (Claus et al., 2012; Kwee & Kwee, 2015; Radhakrishna, Gayathri, & Chegu, 2013). In comparison, liquid biopsy holds huge potential for cancer diagnosis with non‐invasiveness and high sensitivity features by analyzing biomarkers in blood (Ignatiadis et al., 2016; Junqueira‐Neto, Batista, Costa, & Melo, 2019; Krawczyk, Fehm, Banys‐Paluchowski, Janni, & Schramm, 2016; Massihnia et al., 2016; Mathai et al., 2019; Zhang et al., 2019).

Among all the emerging biomarkers, extracellular vesicles (EV) are able to carry multiple functional biomolecules (such as proteins, lipids, RNA and DNA fragments) inherited from their parental cells (Hu et al., 2017, Huang et al., 2019, Kalnina et al., 2015, Liu et al., 2019, Mol, Goumans, Doevendans, Sluijter, & Vader, 2017, Zhu et al., 2019). Due to their high concentration (up to 10^11^ vesicles/ml) and stability in blood circulation (Lee et al., 2018), tumour‐derived EVs are regarded as promising biomarker for liquid biopsies in cancer patients. For instance, quantification of GPC1^+^ circulating exosomes (an extracellular vesicles) in serum has been applied for pancreatic cancer diagnosis (Melo et al., 2015). In another case, detecting of endothelial locus‐1 protein (Del‐1) on circulating EV has been utilized to distinguish breast cancer from benign breast tumours and non‐cancerous diseases (Moon et al., 2016).

Interestingly, EV derived from tumour cells with different subtypes may carry distinct biological information (Bobrie & Thery, 2013, Sandfeld‐Paulsen et al., 2016). For example, a recent study about single‐cell EV secretion has demonstrated that there are significant differences in the quantity and phenotypes of EV secreted by different individual cells (Ji et al., 2019), which suggests that the cancer cell‐derived EV may also be highly heterogenous (Liu et al., 2019, Smith et al., 2015). By profiling the surface proteins on individual EV, the EV‐to‐EV heterogeneity information may assist in developing cancer diagnostic methods.

Conventional methods (such as proteomic, Western Blot and ELISA) for EV analysis are mainly based on bulk measurements, which masks the differences between individual EV (Gangoda et al., 2017, Im et al., 2014, Shao et al., 2012). Recently, some advanced methods have been developed for single EV analysis (Liu et al., 2018, Wang et al., 2019, Yang et al., 2018). For example, a droplet digital ExoELISA assay has been utilized to achieve absolute counting of single exosome in plasma with a limit of detection (LOD) down to 10 exosomes per microliter, and is able to distinguish breast cancer patients before and after surgery (Liu et al., 2018). K. Lee et al. developed a single EV analysis (SEA) technique using repeated stain imaging method and measured 11 different protein markers in a single vesicle (Lee et al., 2018). However, because of the small size and low expression level of surface proteins, advanced imaging technique is needed for individual EV analysis. Recently, D. Wu used proximity barcoding assay to amplify the protein signal on individual exosomes and achieved surface protein analysis on individual exosomes (Wu et al., 2019). But the potential of individual EV heterogenity analysis for clinical sample measurement has not been fully explored.

In this work, we propose an ultrasensitive assay of digital profiling of proteins on individual EV (DPPIE) for sensitive tumour diagnosis and classification using clinical plasma samples. Specifically, we firstly conjugated anti‐CD9 antibodies to the surface of a biochip, and all the CD9^+^ EV can be captured and enriched on the biochip. Secondly, the CD63 DNA aptamer was added into the biochip to recognize CD63 protein on EV. Comparing with antibodies, DNA aptamers have more stable performance and can be directly used to trigger downstream signal amplification reaction. Using anti‐CD9 antibody and CD63 DNA aptamer, we constitute a double‐check strategy to eliminate the interference from soluble molecules and ensure that the detection is specific for EV. Simultaneously, aptamers of EpCAM (various cancers) (Kimura et al., 2010, Kimura et al., 2007, Konigsberg et al., 2011, Sen & Carnelio, 2016) and MUC1 (adenocarcinoma) (Croce et al., 2003, Jing, Liang, Hao, Yang, & Cui, 2019, Saltos et al., 2018) were added into the biochip to recognize the specific surface proteins on EV. Then, a rolling circle amplification (RCA) reaction (an isothermal, enzymatic reactions mediated by Phi29 DNA polymerases to generate long and repeated single stranded DNA molecules) (Deng et al., 2018, Zhao, Ali, Brook, & Li, 2008) was applied to generate localized amplified fluorescent signals on each of EV for ultrasensitive digital detection and multiprotein profiling. We have proved that DPPIE assay is able to analyze the heterogeneity of individual EV from 30 μl diluted plasma without purification steps. Using DPPIE, we are able to distinguish multiple cancer types (breast, lung and leukaemia) and differentiate lung adenocarcinoma patients from lung squamous carcinoma patients by sensing the heterogenous protein expression of individual EV using more than 60 clinical samples. More importantly, we found that the high‐dimensional heterogeneity analysis of individual EV could provide more information for multi‐cancer diagnosis and classification.

## 2 MATERIALS AND METHODS

### 2.1 Materials and reagents

Oligonucleotide sequences purified by HPLC were obtained from Sangon (Shanghai, China) and the sequences were provided in Table S8. Non‐interference protein assay kit was also obtained from Sangon (Shanghai, China). Cover glasses (24 × 50 mm), T4 DNA ligase, 10 × T4 DNA ligase reaction buffer, phi29 DNA polymerase and 10 × phi29 DNA polymerase reaction buffer were bought from Thermo Scientific (Waltham, USA). Adenosine 5'‐triphosphate (ATP), RNase A and deoxynucleotide (dNTP) solution mix were purchased from New England Biolabs (NEB, USA). Polydimethylsiloxane (PDMS, Sylgard 184 silicone elastomer kit) and curing agent were obtained from Dow Corning (USA). ExoQuick‐TC Exosome Isolation Reagent and Exosome‐depleted FBS were provided from System Biosciences (SBI, USA). Syringe‐driven filter unit (0.22 μm), 100 kDa MWCO centrifugal ultrafiltration tube and polyvinylidene difluoride (PVDF) membranes were bought from Millipore (USA). Streptavidin, 1‐ethyl‐3‐(3‐dimethylaminopropyl) carbodiimide hydrochloride (EDC•HCl), N‐hydroxysuccinimide (NHS), 4‐(2‐hydroxyethyl)piperazine‐1‐ethanesulfonic acid (HEPES), salmon sperm DNA, RPMI medium 1640, 3‐aminopropyltriethoxysilane (APTES), 2‐morpholinoethanesulfonic acid monohydrate (MES), 1 kb plus DNA ladder and SolarGel Red Nucleic Acid Gel Stain (10000 × ) were acquired from Solarbio Life sciences (Beijing, China). Exosomal protein extraction kit was purchased from 101 Bio (USA). And anti‐CD9, anti‐CD63, anti‐EpCAM and anti‐MUC1 were provided by Abcam (UK). Mouse anti‐Rabbit IgM/Alexa Fluor 488, rabbit anti‐CD9/AF488 conjugated antibody and anti‐CD9/biotin were obtained from Bioss Antibodies (Beijing, China). Human ELISA kits (CD63, EpCAM and MUC1) were acquired from AMEKO (Shanghai, China). Antifade mounting medium and RIPA lysis buffer were bought from Beyotime (Beijing, China). DMEM/high glucose medium was purchased from Hyclone (Beijing, China). Series S Sensor Chip SA and 10 × HBS‐EP+ buffer were purchased from GE Healthcare (Uppsala, Sweden). And the other reagents were analytical grade and all solutions were prepared using deionized water with Milli‐Q water (18 MΩ).

### 2.2 Cell culture

Human breast adenocarcinoma cells MCF‐7, human normal breast epithelial cells Hs578Bst,  murine melanoma cells B16F10 and mouse mononuclear macrophages cells RAW264.7 were provided by Chinese Academy of Sciences Cell Bank. The MCF‐7, Hs578Bst and B16F10 cells were cultured in RPMI medium 1640 with 10% Exosome‐depleted FBS and 1% penicillin/streptomycin. The RAW264.7 cells were maintained in DMEM/high glucose medium with 10% exosome‐depleted FBS and 1% penicillin/streptomycin. All cells were cultured at 37 °C in a humidified incubator containing 5% CO_2_ and supernatants were gathered when the cells reached 80–90% confluence.

### 2.3 EV preparation

EVs were isolated as described previously (Zhang et al., 2018). In brief, supernatants were centrifuged at 3,000 × *g* for 15 min at 4 °C and then filtered by a syringe‐driven filter unit (0.22 μm) to separate cells and cells debris. Next, the sample was treated by ultrafilter at 5,000 × *g* for 15 min at 4 °C using 100 kDa MWCO. Subsequently, the above ultrafiltrates were mixed with ExoQuick‐TC solution and incubated overnight at 4 °C. After centrifugation (5,000 × *g*, 4 °C and 30 min), supernatants were discarded and the EV pellet was resuspended in PBS buffer (pH 7.2‐7.4).

### 2.4 EV characterization

The morphology, size and concentration of EV derived from MCF‐7 cells were characterized through Transmission Electron Microscopy (TEM, Tecnai F20, FEI) and Nanoparticle Tracking Analysis (NTA, NS300, Malvern Instruments).

### 2.5 Western Blot

Total proteins from EV were prepared using Exosomal Protein Extraction Kit. And total proteins from cells were extracted by RIPA lysis buffer. Concentration of proteins was measured by non‐interference protein assay kit. Western Blot analysis was conducted using standard method.

### 2.6 Preparation of PDMS stamps

PDMS stamps were prepared by mixing elastomer with curing agent (Dow Corning Sylgard 184, w/w = 10:1) and heating at 65 °C for 12 h after degassing.

### 2.7 Preparation of engineered biochip

Amino‐modified coverslips ‐were firstly fabricated. Briefly, coverslips (24 × 50 mm) were immersed in piranha solution (mixture of 98% H_2_SO_4_ and 30% H_2_O_2_ with v/v = 7:3) for 1 h and following the coverslips were soaked with a 5% APTES solution in methanol for 12 h at room temperature (RT). Then the above coverslips were washed with Milli‐Q water and dried under nitrogen gas. Next, a biochip was prepared using amino‐modified‐coverslip enclosed by PDMS gelatine (20 × 45 mm) with chambers (4 mm in diameter). The substrate of biochip was further functionalized with streptavidin by immersed in solution [50 mM MES (pH 5.5), 200 mM EDC, 100 mM NHS, and 100 μg/ml streptavidin] for 1.5 h at RT, then rinsed with PBS to remove uncombined streptavidin. Next, added 1 μg/ml anti‐CD9/biotin into biochip and incubated for 1 h at RT, then rinsed with PBS to remove free anti‐CD9/biotin.

### 2.8 Characterization of anti‐CD9 functionalized biochip

To verify engineered biochip successfully conjugated biotin‐labelled anti‐CD9, 1 μg/ml mouse anti‐Rabbit IgM/Alexa Fluor 488 was poured into the anti‐CD9 functionalized chamber to incubate for 1 h at RT, following by rinsed with PBS to wash away unreacted secondary antibodies. In addition, untreated coverslip was served as a negative control. Finally, antifade mounting medium was added to prevent fluorescence quenching and the chamber was imaged by a Confocal Laser Scanning Microscope (CLSM, Leica TCS SP8*, Germany).

### 2.9 Preparation of circular DNA templates

Circular DNA templates (20 μl) were prepared by mixed with 2 μl of the phosphorylated padlock probe (10 μM), 2 μl of ligation template (10 μM), 2 μl of 10 × T4 DNA ligase reaction buffer (400 mM Tris‐HCl, 100 mM MgCl_2_, 100 mM DTT, 5 mM ATP), 13.5 μl of DEPC‐treated H_2_O and 0.5 μl of T4 DNA ligase (5 Weiss U/ μl). And mixtures were incubated at RT for 2 h and then heated at 70 °C for 5 min to terminate reaction.

### 2.10 RCA reaction

The RCA reaction was performed in a volume of 20 μl containing 2 μl circular ligation template (1 μM), 2 μl of 10 × phi29 DNA polymerase reaction buffer [330 mM Tris‐acetate, 100 mM Mg‐acetate, 660 mM K‐acetate, 1% (v/v) Tween 20, 10 mM DTT], 2 μl dNTPs (1 mM), 13.5 μl of DEPC‐treated H_2_O and 0.5 μl phi29 DNA polymerase (10 U/ μl). The RCA reaction was performed at 37 °C for 60 min and inactivated by heating at 65 °C for 10 min.

### 2.11 RCA products characterization

RCA products were estimated by agarose gel electrophoresis using agarose gel (1%) for 90 min (90 V), stained with SolarGelRed Nucleic Acid Gel Stain, followed by imaging under Gel Imaging Analysis System (BIO‐RAD, Gel Doc XR^+^). In addition, the size and morphology of RCA products were further characterized by Dynamic Light Scattering (DLS, Zetasizer Nano ZS‐90, Malvern Instruments), TEM and Field Emission Scanning Electron Microscope (FESEM, MERLIN Compact, Carl Zeiss).

### 2.12 EV capture

EVs successfully captured by anti‐CD9 functionalized biochip was elucidated through SEM. Briefly, engineered biochip was blocked with blocking/reaction buffer [20 mM HEPES (pH 6.5), 100 mM NaCl, 5 mM MgCl_2_, 0.1 mM EDTA, 1 mM freshly added DTT, 250 μg/ml BSA, 2.5 μg/ml sonicated salmon sperm DNA, 0.05% Tween 20]. Subsequently, 30 μl of EV were added into chamber of biochip incubation at RT. After 1 h, the chamber was washed 3 times (5 min for each time) with PBS to wash away free EV. Finally, the sample was dried under 30 °C for 12 h and imaged through FESEM.

To estimate the percentage of CD9‐positive (CD9^+^) EV in the whole EV population, a ZetaView PMX120 (Particle Metrix GmbH, Meerbusch, Germany) NTA machine with fluorescence measurement ability was used for the test. Briefly, EV suspensions from MCF‐7 cells were incubated with rabbit anti‐CD9/AF488 conjugated antibody (a final concentration of 5 μg/ml) in the dark at RT for 1 h. Next, EV was measured in scatter mode to define total EV and in fluorescence mode to define CD9^+^ EV.

### 2.13 Kinetic analysis of EV‐aptamer interaction

Kinetics of EV‐aptamer interaction were analyzed using a Biacore T200 optical biosensor (GE Healthcare, Uppsala, Sweden). Specifically, the ligands of biotinylated aptamer_CD63_, aptamer_EpCAM_ and aptamer_MUC1_ were diluted to the concentration of 0.125 μg/ml with 1 × HBS‐EP+ buffer (10 mM HEPES, 150 mM NaCl, 3 mM EDTA, and 0.05% v/v surfactant P20), and then immobilized on streptavidin sensor chip (Series S Sensor Chip SA) at flow rate 5 μl/min on different channels, reaching immobilization levels of 50.5, 55.7 and 53.6 RU, respectively. Then, increasing concentrations of EV (concentration range from 1.25 to 20 nM) in 1 × HBS‐EP+ buffer were injected at flow rate 30 μl/min to associate for 300 s, followed by a dissociation step of 600 s. After each run, the sensor chip was regenerated by injection of 0.5% SDS. All sensor grams were corrected for baseline drift by subtracting a control sensor exposed to running buffer. Kinetic parameters were calculated using a 1:1 binding model in Biacore T200 evaluation software.

### 2.14 Optimization of the concentration of ligation template in preparation EV@RCA

EV@RCA was prepared using standard method of RCA reaction. In short, after equivalent EV in different chambers were captured by engineered biochip, series concentration of ligation template_CD63_ (0, 1, 10, 20, 50, 100, 150 and 200 nM) were added to the chamber and incubated for 30 min at RT, respectively. And then the chamber was washed three times (5 min for each time) with PBS to remove unbounded ligation template_CD63_. Next, RCA reaction was conducted and detection probe_CD63_ was added into chamber. Subsequently, the biochip was incubated for 30 min at RT and washed thoroughly with 1 × TBST buffer (274 mM NaCl, 40 mM Tris‐HCl and 0.1% Tween 20). Finally, antifade mounting medium was poured into the chamber and was imaged by CLSM.

### 2.15 EV@RCA characterization

The morphology and size distribution of EV@RCA was characterized through SEM. In brief, EV@RCA produced in engineered biochip were washed with deionized water, and dried under a gentle N_2_ stream, then the biochip was incised to the right size. Finally, biochip was coated a 3 nm layer of gold using a direct current sputter coating approach and imaged with FESEM.

### 2.16 Investigation whether the molecular crowding would affect the detection

To investigate whether the molecular crowding would affect the detection, we firstly incubated equal amount EV on 3 separate anti‐CD9 modified biochips for 60 min and washed with PBS for three times. Then, for chip 1, we directly added aptamer_EpCAM_ to label EV. While for chip 2 and chip 3, we added aptamer_CD63_ and aptamer_CD63_ + aptamer_MUC1_ respectively and incubated for 30 min before addition of aptamer_EpCAM_. Afterwards, RCA reaction was performed on all 3 chips to prepare EV@RCA_EpCAM_ and imaged with CLSM.

### 2.17 Triple‐protein‐marker‐positive EV@RCA

Experimental procedures were similar to EV@RCA assay of single protein. Moreover, in the preparation of triple‐protein‐marker‐positive EV@RCA, ligation reaction was conducted with three kinds of ligation templates (CD63, EpCAM and MUC1), and three kinds of fluorophore‐labelled detection probes were added into before imaging.

### 2.18 Specificity of DPPIE assay

Specificity was evaluated using EV from MCF‐7, Hs578Bst, B16F10 and RAW264.7 cell lines. Firstly, the concentration and size of prepared EV were quantified by NTA. Secondly, triple‐protein‐marker‐positive EV@RCA was prepared according to above described method, respectively. Finally, antifade mounting medium was added into the chamber before imaging.

### 2.19 Characterization of EV@RCA from cell supernatants

To demonstrate the suitability of DPPIE assay for detecting EV in supernatants, freshly collected MCF‐7 culture medium supernatants were poured into engineered chip. EV were captured and other impurities were discarded by washing, and then imaged using SEM. In addition, EV@RCA from supernatants was also prepared and imaged through CLSM.

### 2.20 Enzyme‐linked immunosorbent assay (ELISA)

ELISA was carried out in accordance with instruction of ELISA Kit. In brief, isolated EV or plasma samples (10^3^ times dilution), bio‐antibody and streptavidin‐HRP were added into 96‐well plates pre‐coated with antibody and incubated at 37 °C, respectively. After 60 min, liquid was discarded and washed completely. Next, chromogen solution was poured into to each well and preservation for 15 min at 37 °C to evade the light. Finally, stop solution was infused to each well and absorbance was measured at 450 nm. Control group was conducted without EV or plasmas. Each sample was measured in three repetitive experiments.

### 2.21 Comparison of DPPIE assay and a commercial available chip

To further demonstrate the sensitivity of DPPIE assay, we bought a surface plasmon resonance (SPR)‐based biosensor chip (Series S Sensor Chip SA, GE Healthcare, Uppsala, Sweden) for comparison, which was mainly based on measuring optical contrast originating from a change in interfacial refractive index due to biomolecular adsorption. BIACORE T200 optical biosensor (GE Healthcare, Uppsala, Sweden) was used for the test. The ligand of biotinylated aptamer_CD63_ was diluted to the concentration of 0.125 μg/ml with 1 × HBS‐EP+ buffer (10 mM HEPES, 150 mM NaCl, 3 mM EDTA, and 0.05% v/v surfactant P20), and immobilized on the biosensor chip, reaching immobilization levels of 50.5 RU. Different concentrations of EV solutions were then injected for 300 s over the aptamer‐functionalized surface. After each run, the sensor chip was regenerated by injection of 0.5% SDS. All sensor grams were corrected for baseline drift by subtracting a control sensor exposed to running buffer only. The molecular fingerprints were obtained by weighting maximal responses with respective aptamer surface coverage and normalized to CD63 responses.

### 2.22 Clinical samples collection

Plasmas were collected from the First Affiliated Hospital of Zhengzhou University. The study was performed complied with the Declaration of Helsinki, and it was approved by the Life Sciences Ethics Review Committee of Zhengzhou University. All participants signed written informed consent before whole blood collection. And samples (*n* = 68) were anonymous, and only the gender, age and pathological diagnosis were recorded. Whole blood samples were collected into EDTA‐coated tubes and mixed gently, followed by centrifugation at 2,000 × *g* for 10 min. Plasma was carefully collected and stored at ‐80 °C before use. Relevant information of plasma samples was shown in Table S9‐S11.

### 2.23 Test of the *‘*dose response’ of plasma sample with different dilution times

To test the *‘*dose response’ of plasma sample with different dilution times, plasma samples with different dilutions were firstly added to the anti‐CD9 coated biochip. Then, SEM was used to characterize the captured EV. Afterwards, RCA reaction was performed on the chip for in situ fluorescent imaging. Finally, the plasma diluted 1000 times was used in the following experiments (according to the Figure S18).

### 2.24 Plasma EV capture and characterization

Next, plasma samples from 68 participants (including 53 cancer patients and 15 healthy donors) were diluted 1,000‐fold with PBS (1 μl of plasma diluted 1,000‐fold). The morphology, size and concentration of particles in plasma were measured by NTA and TEM. Moreover, 30 μl diluted plasma samples were directly added to the anti‐CD9 engineered biochip pre‐blocked with blocking/reaction buffer, and incubation for 1 h at RT, so that all plasma EV that express this common EV marker was captured and enriched. Next, the chamber was washed 3 times (5 min for each time) with PBS to wash away other particles. Finally, the sample was dried under 30 °C for 12 h and imaged through FESEM.

### 2.25 Preparation of ‘spike‐in’ EV with different concentration in plasma

Plasma sample was centrifuged at 100,000 × *g* overnight at 4 °C, and supernatants were collected as non‐EV containing plasma. EV derived from MCF‐7 cells with known concentration were spiked in the non‐EV containing plasma to prepare plasma samples with series of EV concentration (2.32 × 10^5^, 2.32 × 10^4^, 2.32 × 10^3^, 2.32 × 10^2^ and 2.32 × 10^1^ particles/μl).

### 2.26 Enzymatic treatment

To investigate whether miRNAs in plasma affect the RCA amplification reaction, we performed a control experiment using RNase A to treat plasma sample before test. Briefly, EV from plasma sample were firstly captured on the biochip, then treated with 10 ng/μl RNase A (New England BioLabs, NEB) for 15 min at 37 °C (de Jong et al., 2020; Endzelins et al., 2017), followed by aptamer labelling and RCA reaction to generate EV@RCA.

### 2.27 Stability of plasma EV@RCA

To investigate the stability of EV@RCA, plasma EV were captured by engineered biochip, and EV@RCA was prepared and imaged using CLSM after storage for 0, 7, 14 and 21 days, respectively. In addition, to further investigate the effect of light, EV@RCA was illuminated for 1 h in duration with a UV source (254 nm, 6 W) before CLSM imaging. Untreated with UV source was as control group.

### 2.28 DPPIE assay for detecting plasma EV

Plasma EV (diluted 1000‐fold of 68 plasma samples) were captured by anti‐CD9 functionalized biochip, and EV@RCA was prepared, separately. Plasma EV@RCA products were imaged by CLSM, respectively.

### 2.29 Statistical analysis

The fluorescence images of EV@RCA amplicons were acquired with a Leica TCS SP8^*^ inverted Confocal Laser Scanning Microscope (CLSM, Leica, Germany) with a 63 × oil‐immersion objective. Images were collected as z stacks with a distance of 0.15 μm between the z slices ‐ to ensure that all EV@RCA amplicons were imaged. The number of EV@RCA in per frame was counted through Image J software. A t‐Distributed Stochastic Neighbor Embedding (tSNE) algorithm was applied for reducing the dimensionality of complex data. tSNE was calculated with EZKit (Version 1.0, EZKit LLC. USA) and the algorithm was based on the research of Laures van der Maaten and Geoffrey Hinton (van der Maaten & Hinton, 2008). The parameters of details were as follows: perplexity: 30; number of iterations: 1,000; momentum: 0.5; learning rate: 200. The algorithm in this study was attached Table S1. Figures were prepared using SigmaPlot version 12.5 (SigmaPlot), Origin version 8.5 software (Origin) and GraphPad Prism version 7.0 (GraphPad). Significance analyses were performed using IBM SPSS Statistics version 19.0 by one‐way ANOVAs. Differences with *P* < 0.05 were considered statistically significant. Receiver operating characteristic (ROC) curves were used to determine diagnostic accuracy, which was prepared using MedCalc statistical software. All data points derived from each experiment ‐was repeated at least three times.

## 3 RESULTS AND DISCUSSION

### 3.1 Overview of DPPIE assay

**FIGURE 1:**
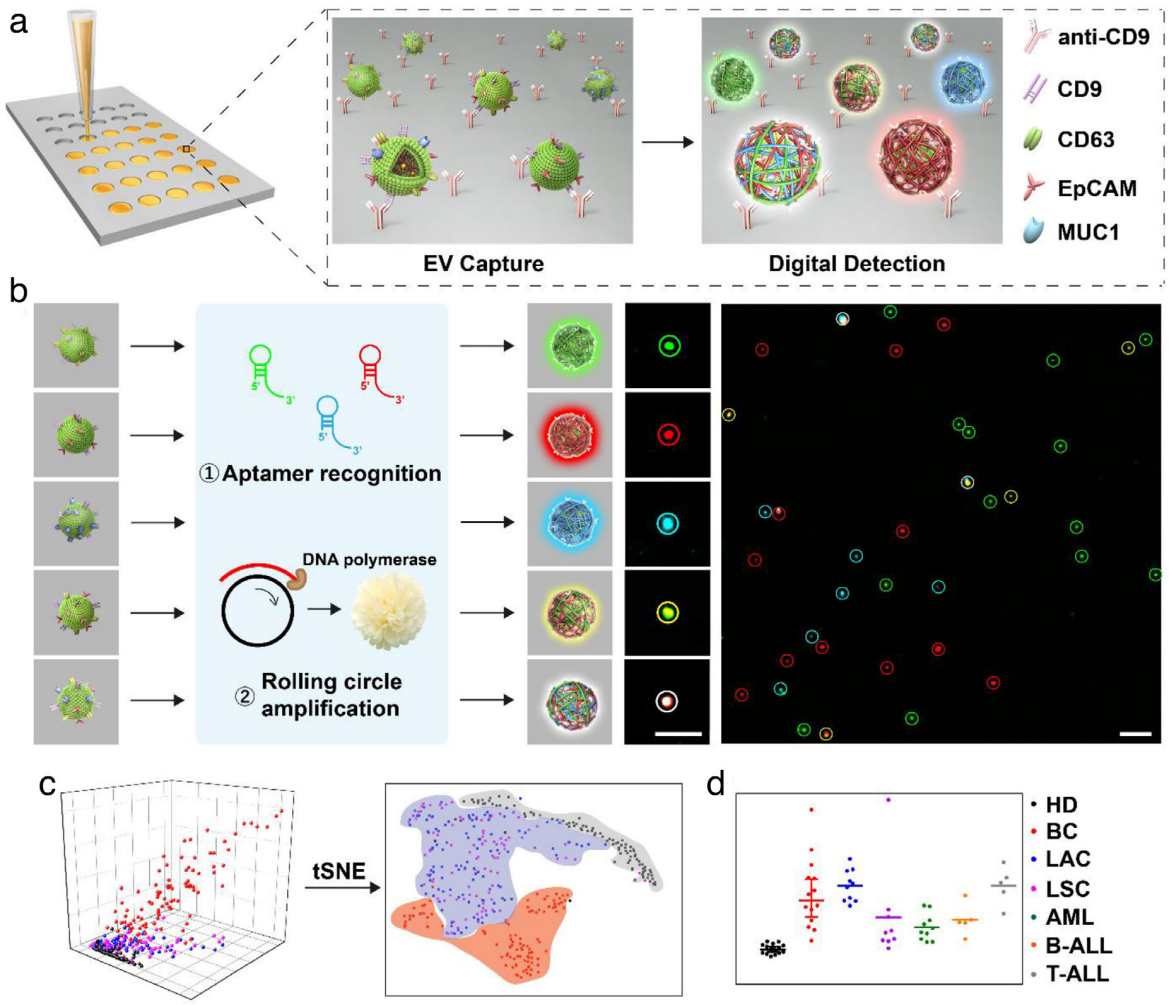
Schematic diagram of digital profiling of proteins on individual EV (DPPIE) for high‐dimensional individual EV analysis and multi‐cancer classification. (a) Overview of the steps in DPPIE assay. Plasma EV is captured on the surface of anti‐CD9 engineered biochip. EV with various surface proteins shows different signals. (b) The captured EV is labelled with DNA aptamers, followed by RCA to generate localized amplified fluorescent signals that can be imaged by confocal microscopy. Fluorescence images showed CD63 (green), EpCAM (red) and MUC1 (blue) expression on individual EV. Scale bar: 3 μm. (c) Individual EV analysis data from different cells are classified using tSNE algorithm. (d) Multi‐cancer diagnosis and classification

The mechanism of DPPIE assay conducted in a biochip was illustrated in Figure 1. Diluted plasma samples of 30 μl (1 μl of plasma diluted 1,000‐fold) were directly added to the anti‐CD9 engineered biochip, and then the CD9^+^ EV was captured (Figure 1a). This immobilization steps help to keep EV spatially fixed on the surface of biochip and facilitated washing steps. The captured EV ‐was then treated with multiple DNA aptamers for specific recognition of EV membrane proteins. Subsequently, RCA reaction was conducted using DNA aptamers as primers to produce long single‐stranded DNA. The corresponding fluorophore‐labelled detection probes were then added to stain the RCA product, which would present bright fluorescent spots under confocal microscopy. Accordingly, CD63, EpCAM and MUC1 proteins on individual EV were designed to present green, red and blue fluorescence respectively. When EV contained both CD63 and EpCAM, their corresponding probes would be coupled, giving yellow spots. Similarly, white spots resulted from the co‐existence of green, red and blue (Figure 1b). A t‐distributed stochastic neighbour embedding (tSNE) algorithm was applied to analyze the subpopulation signature of individual EV from different cells with varied expression of CD63, EpCAM and MUC1 (Figure 1c). Furthermore, the analysis of surface protein heterogeneity on individual EV enable identification of different kinds of tumours using clinical plasma samples (Figure 1d).

### 3.2 DPPIE assay for ultrasensitive digital detection of EV

**FIGURE 2:**
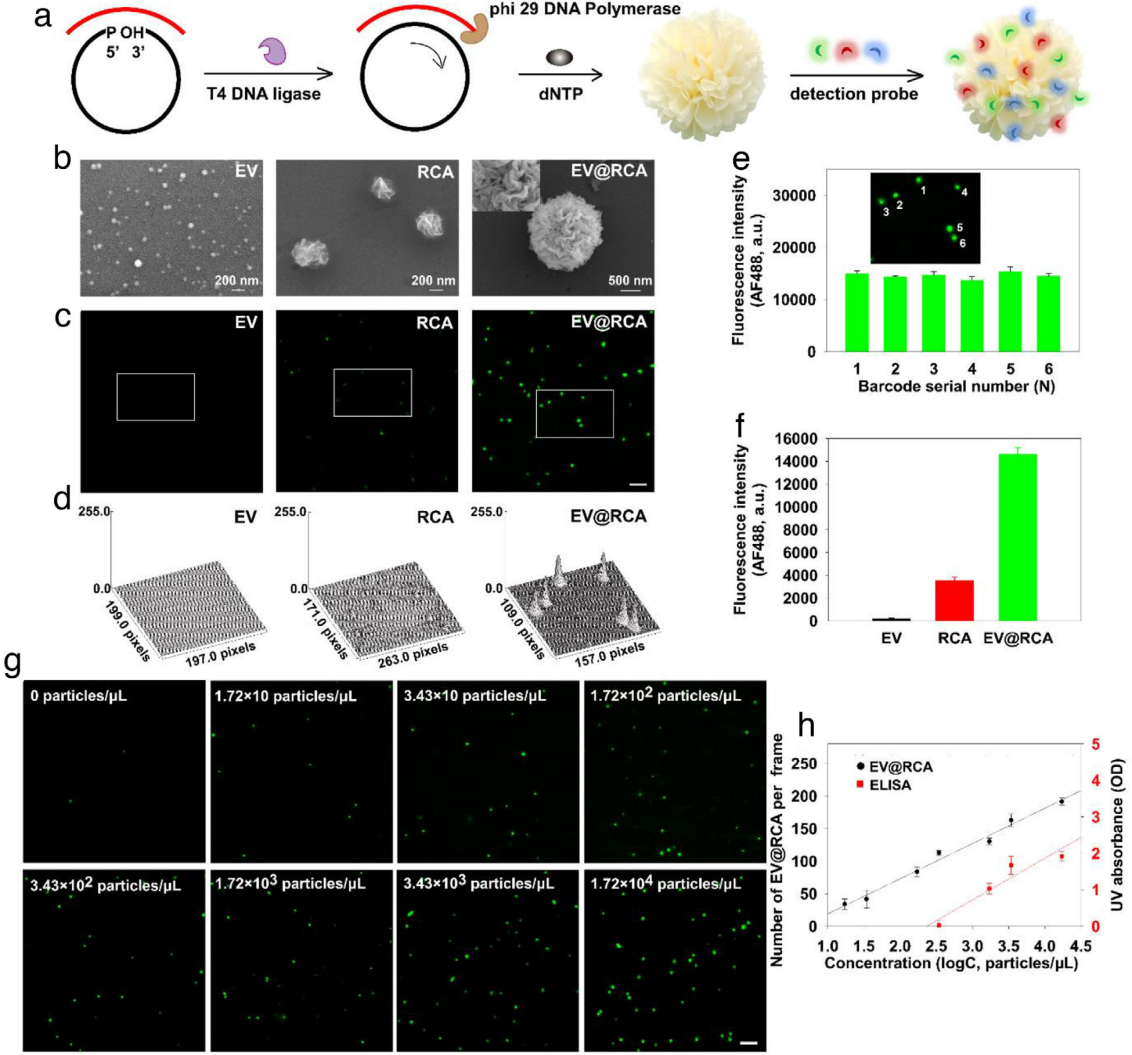
DPPIE assay for ultrasensitive detection of EV from MCF‐7 cells. (a) Schematic illustration of the RCA reaction for generating amplified fluorescent signals. (b) Representative SEM images of captured EV (diameter: 30–120 nm) by anti‐CD9 engineered biochip, RCA products (diameter: ∼300 nm) adhered to the biochip by charge adsorption, and large‐sized “flower” structure of EV@RCA (diameter: ∼2 μm). The top left inset showed a high magnification view of EV@RCA. (c) Representative fluorescent images of EV, RCA and EV@RCA complexes. Scale bar: 3 μm. (d) Fluorescence intensity of EV, RCA and EV@RCA complexes of the marked area in C. (e) Fluorescence intensity of EV@RCA complexes 1–6 in the marked area. (f) The average fluorescence intensity of EV, RCA and EV@RCA complexes. (g) Detection of EV in different concentrations using DPPIE assay. Scale bar: 3 μm. (h) Comparison of the sensitivity between DPPIE and bulk measurement (ELISA) for EV detection. DPPIE is around 100 times more sensitive than ELISA. The LOD of DPPIE is around 10 EV/ μl. Error bars show the standard deviation of triplicate experiments.

To evaluate the performance of the DPPIE assay for EV detection, EV from human breast cancer cell line MCF‐7 were selected as a model. Specifically, the EV were isolated and characterized carefully before use. TEM and NTA analysis showed that the average diameter of isolated EV were approximately 90 nm (Figure S1A‐B). Immunoblotting analysis demonstrated that the CD9 and CD63 proteins were abundantly expressed in the isolated EV but barely in their parent cells (Figure S1C). Since CD9 was a well‐known tetraspanins membrane protein marker of EV, anti‐CD9 antibody was chosen to capture the EV in this study. We have confirmed that the surface of engineered biochip was uniformly coated with anti‐CD9 antibodies (Figure S2) and the EV expressing this EV marker can be captured and enriched (Figure 2b). We also measured CD9^+^ EV, which approximately account for 81.25% of all the EV (Figure S3A‐C and Table S2).

After immoblization, the EV were recognized by different DNA aptamers, and the primers at the 3’‐ end of DNA aptamers were used to initiate RCA reaction to generate long single‐stranded DNA. The principle of RCA reaction was illustrated in Figure 2a. We firstly confirmed the RCA products of long single‐stranded DNA have been generated (Figure S4A and Figure S5). The RCA products formed were further characterized by DLS, TEM and SEM (Figure S4B‐D). It's shown that the RCA products were generally monodisperse particles with around 300–400 nm diameter and petal‐like structures.

Further, kinetics of EV‐aptamer interaction were measured, and the affinity constant (K_D_) of EV for aptamer_CD63_, aptamer_EpCAM_ and aptamer_MUC1_ was 1.76 nM, 0.81 and 3.86 nM, respectively, indicating that EV displays a strong affinity for above aptamers (Figure S6). RCA on EV was then performed. Similar ‘nanoflower’ structures of RCA amplicons were observed on biochip (Figure 2b), indicating that RCA reaction can be conducted on biochip. Since each of EV was recognized by multiple aptamers, the EV were encapsulated by numerous long single‐stranded DNA (RCA products) to form a large‐sized ‘flower’ structure (approximately 2 μm), named EV@RCA. In this work, the digitally quantified amount of EV@RCA was regarded as the number of EV. For fluorescent imaging, EV@RCA_EpCAM_ products at different reaction time were also investigated, and reaction time of 60 min was used in the following experiments (Figure S7). Next, the fluorophore‐labelled detection probes were added to the EV, RCA and EV@RCA samples, respectively. As shown in Figure 2c, no obvious fluorescent signals were found on the EV, and only faint signals were observed on RCA products. In contrast, there were bright spots in the EV@RCA sample. The fluorescent intensity analysis (Figure 2d‐e) indicated that the signals from different EV@RCA were stable and consistent. The average fluorescent intensity of EV@RCA was approximately 65 times higher than fluorescent aptamer labelled EV and 5 times higher than single RCA products (Figure 2f).

Thereafter, we optimized the RCA reaction (Figure S8) and employed DPPIE assay to measure EV with a series of concentrations. It is shown that the number of EV@RCA per frame increased with the EV concentration (Figure 2g), with a linear range of 10 to 10^4^ particles/μl and linear correlation of R^2^ = 0.98, and we achieved a limit of detection (LOD) of 10 EV/μl. The fluorescent intensity of single EV@RCA produced in different concentrations of EV was uniform (Figure S9). Meanwhile, the same set of samples were measured by an enzyme‐linked immunosorbent assay (ELISA) as a head‐to‐head comparison and ELISA failed to measure EV with concentration lower than 10^3^ particles/μl. Further, we bought another surface plasmon resonance (SPR)‐based biosensor chip to compare with DPPIE. As shown in Figure S10, a good linear relationship between RU and EV concentration was obtained (one RU was equivalent to one picogram per square millimetre on the sensor surface), and we achieved a LOD of 1.76 × 10^5^ particles/μl, which was much higher than DPPIE. Indeed, either ELISA or this biosensor chip was based on bulk measurement of total EV, and cannot be applied for analysis of multiple proteins on individual EV. The above results collectively proved that DPPIE assay was able to digitally counting individual EV with ultra‐high sensitivity.

The stability of EV@RCAs was then tested. After exposed in the illumination of UV for 1 h or stored at 4 °C for 21 days, the fluorescence intensity of single EV@RCA had no obvious change (Figure S11‐12). DPPIE assay for analysis of EV in fresh cell culture media was also investigated, showing good performance for detection of EV without isolation (Figure S13A‐B). The photostability and biostability of EV@RCA, integrated with the superior sensitivity for EV detection, provide the basis of DPPIE assay to be applied in profiling of proteins on individual EV, especially in complex biological environment such as plasma.

### 3.3 Multi‐protein profiling on individual EV

**FIGURE 3:**
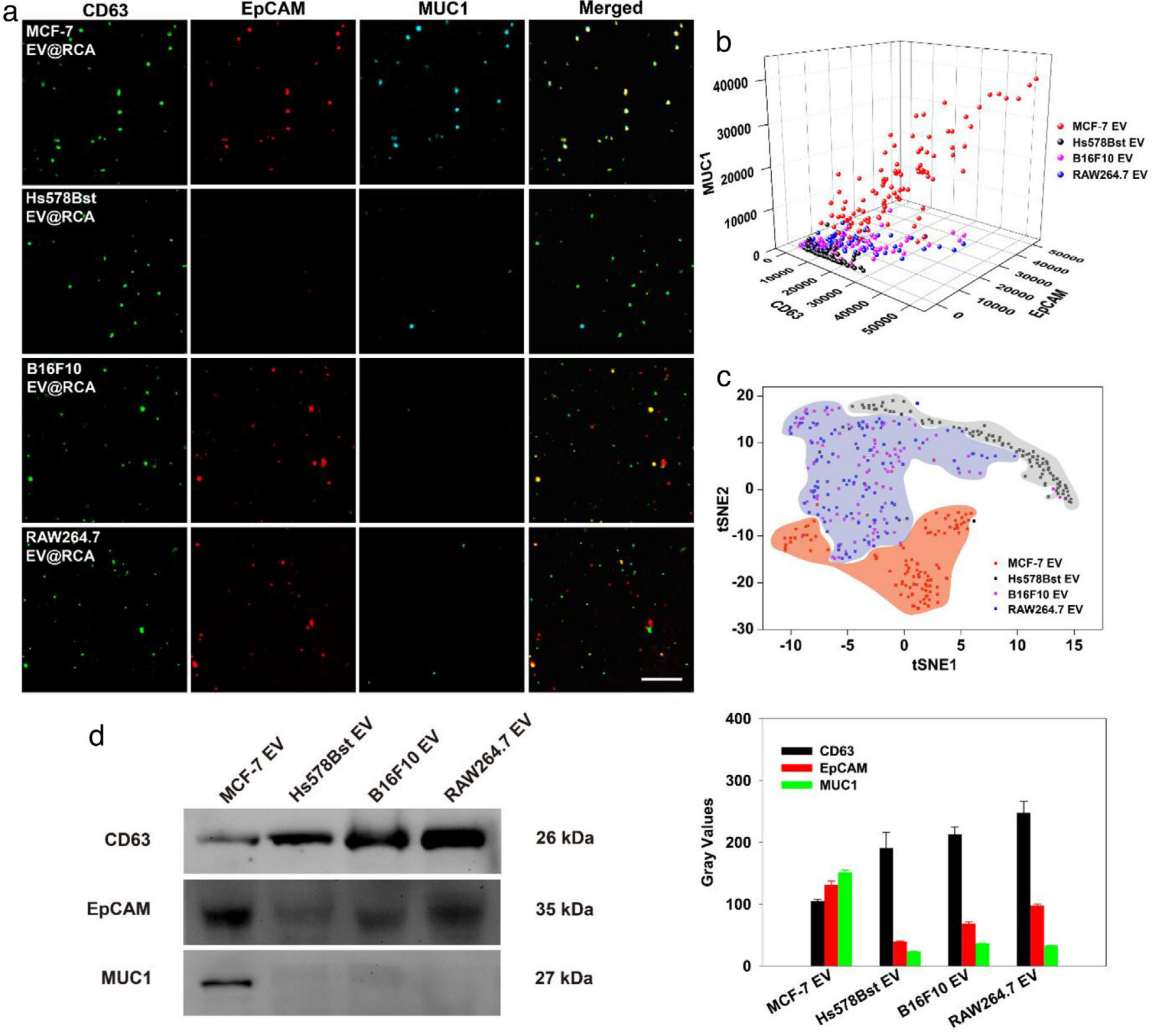
DPPIE assay for multi‐protein analysis on individual EV. (a) Fluorescent images of EV@RCA complexes from EV secreted by human breast adenocarcinoma cells MCF‐7, human normal breast epithelial cells Hs578Bst,  murine melanoma cells B16F10 and mouse mononuclear macrophages cells RAW264.7. Scale bar: 7.5 μm. (b) 3D scatter plots of individual EV information. The fluorescence intensity of CD63, EpCAM and MUC1 were used as XYZ axis in a 3D place. (c) Discrimination of EV secreted by different cell lines through analyzing of the heterogeneous protein expression using two‐dimensional tSNE mapping. To help visualize, clusters were artificially colour coded. (d) Western Blot and semi‐quantitative analysis of the protein expression on EV secreted by different cell lines. Each lane was loaded with 40 μg of total proteins. Error bars show the standard deviation of three replicate experiments.

Taking advantage of the ultrasensitive EV analysis ability of DPPIE, the assay for multiple proteins profiling on individual EV was conducted. We first confirmed that the RCA reaction for different protein detection did not interfere with each other (Figure S14A‐B). Further, we investigated whether the molecular crowding would affect the detection. As shown in Figure S15, the binding of aptamer_CD63_ and aptamer_MUC1_ before aptamer_EpCAM_ did affect the binding of EpCAM probes a little bit, but not in a very significant way (*P* > 0.05). Then, DPPIE assay for simultaneous detection of CD63, EpCAM and MUC1 on individual EV was tested, and super‐bright spots (green, red and blue) were observed in the fluorescent images (Figure S16). Next, EV secreted by different cell lines were measured using DPPIE assay. As shown in Figure 3a, the fluorescent intensity of green, red and blue spots represented the corresponding target proteins expression of CD63, EpCAM and MUC1 on individual EV. The surface protein profiling results demonstrated that the three chosen target proteins were present on the membrane of EV secreted by MCF‐7 cells. In comparison, EV secreted by Hs578Bst cells showed high expression level of CD63 and very low expression of EpCAM and MUC1. Meanwhile, the target proteins presented on EV from B16F10 and RAW264.7 cells were similar to each other, displaying abundantly expression of CD63 and EpCAM, but a fairly low expression of MUC1. These observations were consistent with Western Blot results (Figure 3d). The concentration and size of EV from different cell lines (MCF‐7, Hs578Bst, B16F10 and RAW264.7 cell) were measured using NTA (Figure S17 and Table S3). When using 3D scatter plots to profile the protein expression on individual EV, we were not able to clearly distinguish different EV categories (Figure 3b). But after two‐dimensional tSNE analysis (Figure 3c), the different communities could be more clearly distinguished. According to the results, by analyzing the heterogeneity of individual EV, DPPIE was able to distinguish the individual EV secreted by different cell lines, which can reduce the crosstalk of each sample to improve the measurement specificity.

### 3.4 DPPIE assay for clinical multi‐cancer diagnosis and classification

To test the clinical applicability of DPPIE, we collected plasma samples from 53 cancer patients and 15 healthy donors with permission. Firstly, we tested the *‘*dose response’ of plasma sample with different dilution times and confirmed plasma samples diluted 500 or 1,000 times were more suitable for individual EV analysis (Figure S18A‐B). In this study, the plasma samples with 1,000 times dilution were used in the following experiments. Next, the concentration and size of particles in different plasma samples were analyzed using NTA (Figure S19‐22, Table S4‐S7). TEM was used to further characterize the size and morphology of particles in diluted plasma samples. Results (Figure S23A‐B) showed that particles were in wide range of size distribution (30–450 nm), since the plasma samples probably contained not only EV, but also other particles including microvesicles (50–2000 nm), chylomicrons (75‐1200 nm) and very low density lipoprotein (27–60 nm) (Akers, Gonda, Kim, Carter, & Chen, 2013, Freitas et al., 2019, Mork et al., 2017). After adding samples to anti‐CD9 functionalized biochip, the captured EV presented a narrow size distribution, which may attributed to the removal of other particles or biomolecules in plasma via washing steps (Figure S23C). Notably, the capture process using engineered biochip, rather than extracting EV from plasma, was able to reduce the occurrence of EV degradation or contamination. Then we further confirmed that miRNA in plasma do not have obvious influence on RCA amplification (Figure S24).

Besides, we have prepared non‐EV containing plasma control to demonstrate that DPPIE can specifically detect EV from plasma. As shown in Figure S25, negligible bright fluorescent spots of EV@RCA_EpCAM_ can be observed in the non‐EV containing plasma. While the regular plasma sample showed lots of fluorescent signals. A linear detection of the tumour‐derived EV in plasma sample was then measured. As shown in Figure S26, the number of EV@RCA per frame increased with the spike‐in EV concentration, and a good linear relationship between number of EV@RCA per frame and known EV concentration can be obtained. The correlation equation was Y=71.2X−69.823 with a correlation coefficient of R^2^ = 0.97 (*n* = 3), and we achieved a LOD of 37 EV/μl.

**FIGURE 4:**
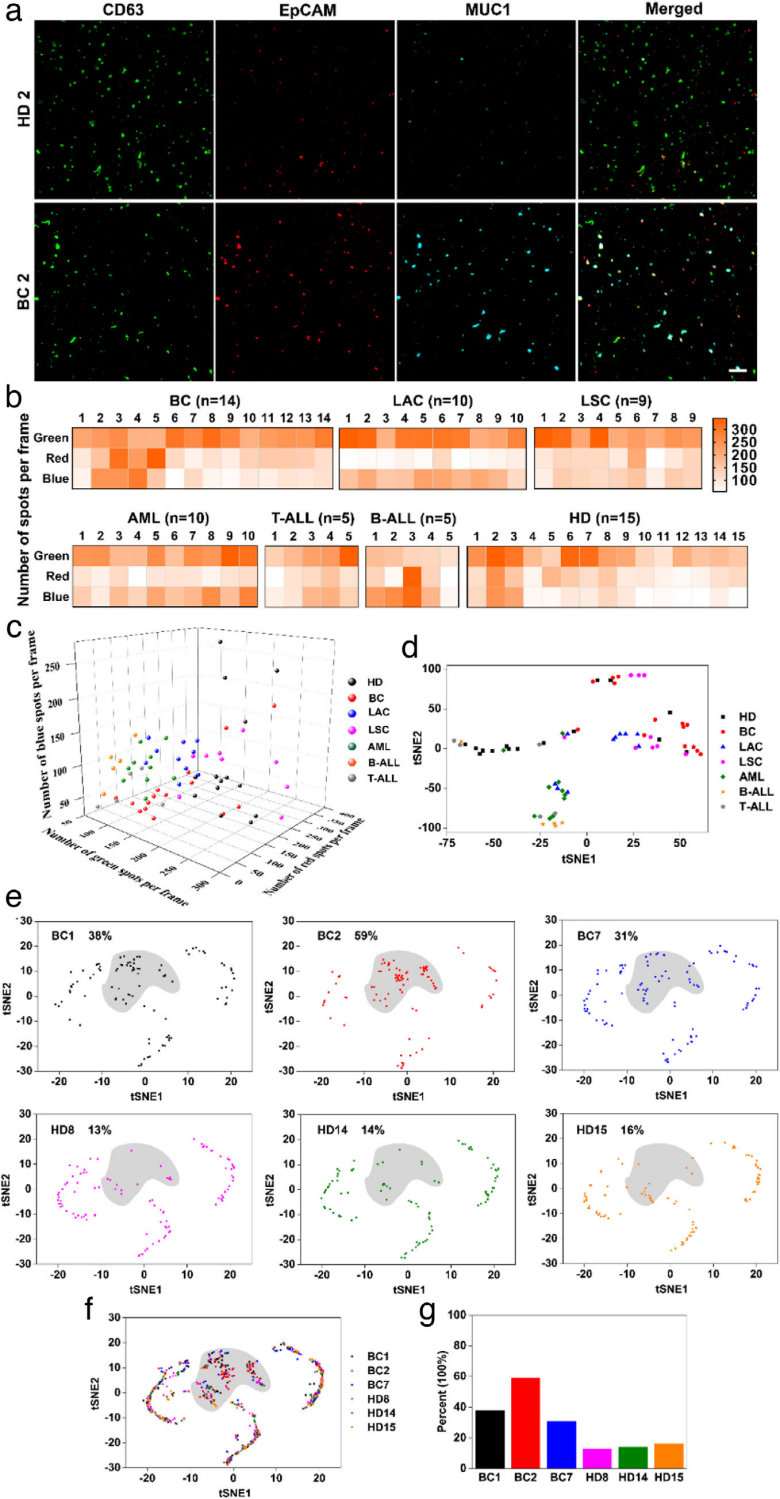
Individual EV heterogeneity analysis improves the discrimination between breast cancer (BC) patients and healthy donors (HD). (a) Fluorescent images of EV@RCA complexes from two clinical samples. Scale bar: 7.5 μm. (b) Heatmap of digital counting analysis for measurements of the number of EV per frame in each clinical plasma sample [lung adenocarcinoma (LAC), lung squamous carcinoma (LSC), acute myeloid leukaemia (AML), T‐cell acute lymphoblastic leukaemia (T‐ALL) and B‐cell acute lymphoblastic leukaemia (B‐ALL)]. (c) 3D scatter plots of specific EV numbers in different clinical samples. (d) Two‐dimensional tSNE mapping of different clinical samples by counting the number of green, red and blue spots in each sample. (e) Individual EV analysis in different clinical samples. 100 individual EV were analysed in each sample. A positive gate representing the main clustering of tumour specific scatter dots was artificially marked with grey area. (f) Overall distribution of individual EV in six clinical samples. (g) The percentage of scatter plots in the positive gate from each clinical sample

Subsequently, the plasma samples were then analyzed using DPPIE assay. Figure 4a exhibited the fluorescent images of EV@RCA in two clinical samples. Other images were shown in Figure S27‐30. The heatmap and 3D scatter plots (Figure 4b‐c) were used to show the calculated number of EV@RCA per frame in each sample. The amount of green, red and blue spots represented the number of EV with high CD63, EpCAM, and MUC1 expression, respectively. According to the results, EV from healthy donors and multi‐cancer (breast, lung and leukaemia) patients could not be distinguished well by counting the number of specific EV. Besides, two‐dimensional tSNE mapping (Figure 4d) was utilized to visualize and classify the EV from healthy donors and cancer patients. Results showed that there were no distinct clusters between each groups, which may be due to the EV secreted by normal cells masking the information of tumour‐derived EV, indicating that bulk measurement may not be able to provide adequate information for EV analysis.

To achieve single EV heterogenity data, DPPIE was applied to analyze the high‐dimensional information of protein expression on individual EV using six plasma samples. The protein profiling data was shown in 3D scatter plots (Figure S31). By analyzing the tSNE mapping of each sample of 100 individual EV, we found that the main difference of scattering dots between breast cancer patients and healthy donors clustered in a defined position (marked in grey), which was mainly corresponding to the CD63/EpCAM/MUC1 triple‐positive EV. Besides, we have estimated 500 individual EV in HD14 and BC2 samples and found that the major difference also comes from the triple‐positive EV (Figure S32). The percentage of EV located in the positive gate showed significant difference between breast cancer patients and healthy donors (Figure 4g), suggesting that DPPIE was able to distinguish the tumour EV from normal community by analyzing the heterogeneous protein expression of individual EV.

**FIGURE 5:**
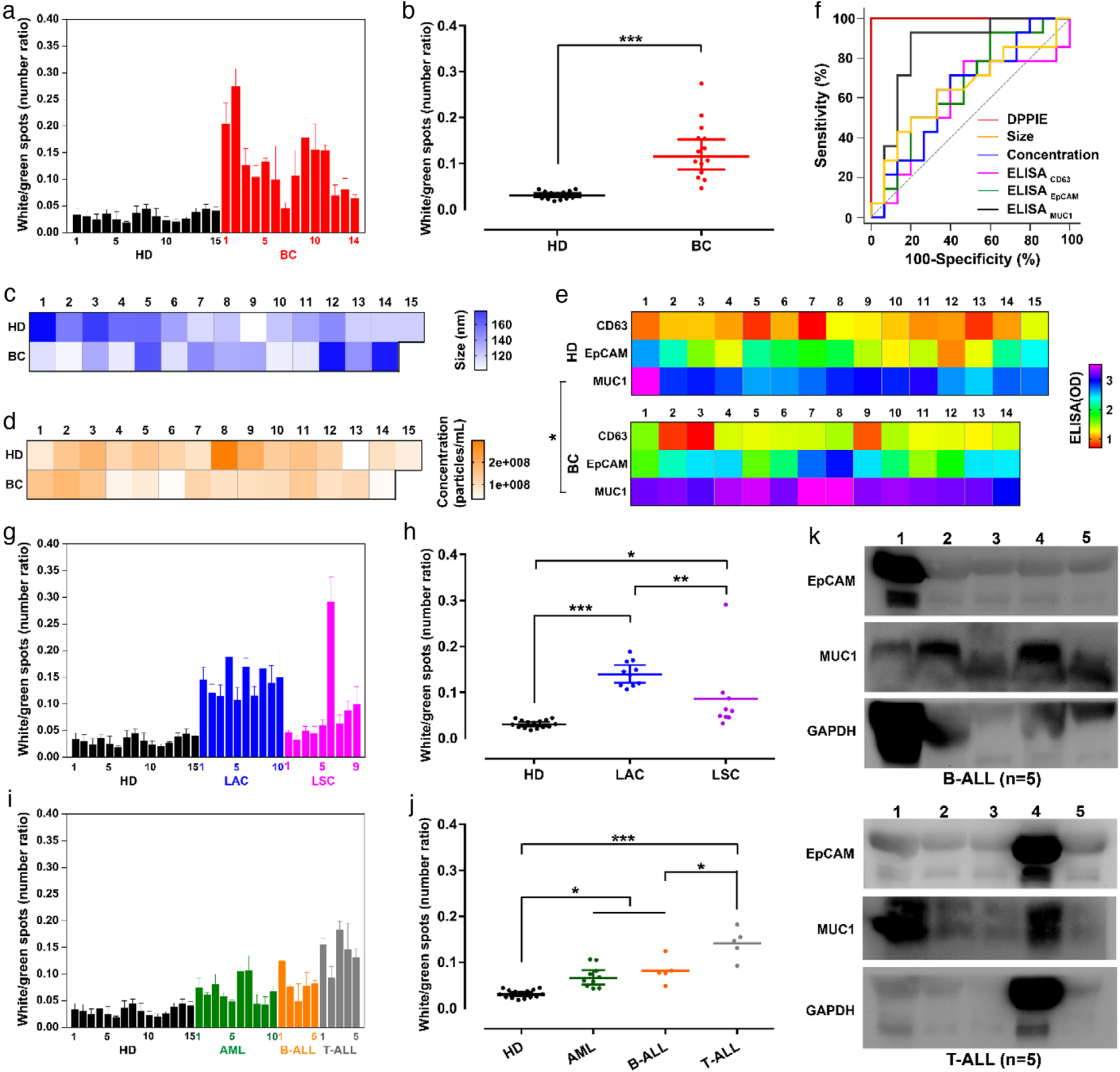
DPPIE assay for detection and classification of multiple type of cancers. (a) Quantification of triple‐positive EV in healthy donors (HD, *n* = 15), and breast cancer patients (BC, *n* = 14). (b) Scatter plots of the white/green spots ratio in the plasma samples of HD and BC. (c) Heatmap of particles size and (d) concentration in the plasma samples from HD and BC. (e) ELISA measurement of the levels of CD63, EpCAM and MUC1 in plasma samples collected from HD and BC. (f) Receiver operating characteristic (ROC) curves between plasma samples from HD and BC, evaluated by DPPIE assay, size, concentration and ELISA, respectively. AUC, area under the curve. (g) Quantification of triple‐positive EV from plasma samples of lung adenocarcinoma (LAC, *n* = 10) and lung squamous carcinoma (LSC, *n* = 9). (h) Scatter plots white/green spots ratio in plasma samples from HD, LAC and LSC. (i) Quantification of triple‐positive EV from plasma samples of acute myeloid leukaemia patients (AML, *n* = 10), B‐cell acute lymphoblastic leukaemia (b‐ALL, *n* = 5) and T‐cell acute lymphoblastic leukaemia (t‐ALL, *n* = 5). (j) Scatter plots of white/green spots ratio in plasma samples from HD, AML, B‐ALL and T‐ALL. (k) Western Blot analysis of EpCAM and MUC1 expression on plasma‐derived EV from patients with B‐ALL and T‐ALL. Error bars show the standard deviation of triplicate experiments. The statistical analysis was performed by one‐way analysis of variance (ANOVA) with post‐hoc Tamhane T2 (* *P* < 0.05, ** *P* < 0.005 and *** *P* < 0.0005)

According to the tSNE mapping of multiple clinical samples, the percentage of CD63/EpCAM/MUC1 triple‐positive EV was used as major parameter for distinguishing different communities. The triple‐ positive EV were corresponding to the white spots, and the number of CD63‐positive EV (green spots) was applied as a control to eliminate the effects of different EV counts. Figure 5a and 5b demonstrated that the ratio of white/green spots in breast cancer (BC, *n* = 14) patients and healthy donors (HD, *n* = 15) have a significant difference (*P* < 0.0005). The receiver operating characteristic (ROC) curve of DPPIE showed an area under the curve (AUC) of 1.0 in breast cancer patients compared to healthy donors, with a specificity and sensitivity of 100% and overall accuracy of 91% (Figure 5f). In comparison, ELISA was inferior in distinguishing BC patients and HD (AUC_CD63_ = 0.595, AUC_EpCAM_ = 0.657 and AUC_MUC1_ = 0.843) (Figure 5e‐f), which may attributed to the bulk measurements feature of ELISA. Besides, neither the size (Figure 5c) of EV nor their concentration (Figure 5d) was an effective parameter to distinguish BC patients and HD (AUC_Size_ = 0.645 and AUC_Concentration_ = 0.633) (Figure 5f). Moreover, univariate logistic regression model also suggested that DPPIE was able to distinguish BC patients and HD in a more efficient way (Figure S33). Although tumour cells were supposed to secrete more EV than normal cells (Zhu, Li, Yang, & Pang, 2018), this subtle distinction could not be seized by NTA measurements.

After applied for carcinoma diagnosis, the ability of DPPIE for classification of tumour subtypes were investigated. The lung adenocarcinoma (LAC, *n* = 10) and squamous carcinoma (LSC, *n* = 9) patients samples were selected as a model. Using DPPIE, we found that the ratio of white/green spots in the lung adenocarcinoma were significantly higher than squamous carcinoma (*P* < 0.005), suggesting DPPIE assay for individual EV analysis may help to facilitate classification of cancer subtypes (Figure 5g‐h). Besides, the plasma samples of leukaemia, including acute myeloid leukaemia patients (AML, *n* = 10), B‐cell acute lymphoblastic leukaemia (B‐ALL, *n* = 5) and T‐cell acute lymphoblastic leukaemia (T‐ALL, *n* = 5) were analyzed ed using DPPIE assay. As showed in Figure 5i and 5j, the ratio of white /green spots in AML, B‐ALL and T‐ALL patient samples were significantly higher than that of HD group (*P* < 0.05), which may due to the over‐expression of EpCAM and MUC1 on their EV. To verify this hypothesis, the EV from B‐ALL and T‐ALL patient's plasma were isolated, and the EpCAM and MUC1 expression of each sample were analyzed using Western Blot (Figure 5k). Although the overexpression of EpCAM and MUC1 in leukaemia cells has been reported in many studies (Guillaume et al., 2019, Stroopinsky et al., 2013, Yin et al., 2010, Zheng et al., 2017), their abundant expression on EV from patients with B‐ALL and T‐ALL was proposed for the first time. The utility of EpCAM^+^/MUC1^+^ circulating EV as potential biomarker for hematologic malignancies diagnosis will be further investigated and validated in a larger cohort in future.

## 4 CONCLUSION

In summary, a localized fluorescent imaging method (termed Digital Profiling of Proteins on Individual EV, DPPIE) was developed for analysis of multiple proteins on individual EV. The features of DPPIE assay include: (i) utilizing the heterogeneity of individual EV to distinguish multiple cancers (breast, lung and leukaemia), suggesting that the high‐dimensional individual EV heterogeneity analysis may provide more precise information for cancer diagnosis and classification; (ii) using localized fluorescent signal amplification method to light up multiple proteins on individual EV, which greatly improves the sensitivity; (iii) first report that CD63/EpCAM/MUC1‐triple‐positive EV do exist in the plasma samples of patients with B‐ALL and T‐ALL, which could be used as potential biomarker for hematologic malignancies diagnosis. We think DPPIE assay holds huge potential for ultrasensitive multi‐cancer diagnosis and classification in clinical application. Besides, owing to its simplicity and general applicability, DPPIE can be widely applied to detect other biomarkers on EV by simply changing aptamers or antibodies. Notably, apart from their composition, EV would also differ in their size and origin (Willms, Cabanas, Mager, Wood, & Vader, 2018) and it is currently still unclear what proteins or their combination on EV can be efficient biomarkers, which need further studied in the future. While we believe that the heterogeneity analysis of individual EV is the key for studying the biogenesis and biofunction of EV.

## CONFLICTS OF INTEREST

The authors declare no competing financial interest.

## Supporting information



Supplementary information
