# Supplementary material for: Localized fluorescent imaging of multiple proteins on individual extracellular vesicles using rolling circle amplification for cancer diagnosis
Source: J Extracell Vesicles. 2020 Nov 11;10(1):e12025. doi: 10.1002/jev2.12025 (PMC7710127; doi:10.1002/jev2.12025)
Supplement: Supplementary file 1 — Supplementary information [file JEV2-10-e12025-s001.docx]

Supporting Information

**Localized Fluorescent Imaging of Multiple Proteins on Individual Extracellular Vesicles Using Rolling Circle Amplification for Cancer Diagnosis**

*Junli Zhang,^†,#^ Jinjin Shi,^†,#^ Hongling Zhang,^†^ Yifan Zhu,^†^ Wei Liu,^†^ Kaixiang Zhang,^†,^* and Zhenzhong Zhang^†,^**

^†^Henan Key Laboratory of Targeting Therapy and Diagnosis for Critical Diseases, School of Pharmaceutical Sciences, Zhengzhou University, Zhengzhou, 450001, China.

**List of Contents:**

1. Figure S1. Characterization of EV.3
2. Figure S2. Identification the surface of biochip coated with anti-CD9.4
3. Figure S3. NTA analysis of EV in the scatter and fluorescence modes………...……….………………..5
4. Figure S4. Characterization of RCA products6
5. Figure S5. Agarose gel electrophoresis analysis of RCA products at varying reaction time…………….7
6. Figure S6. Kinetic analysis of EV-aptamer interaction……………....………………………………..... 7
7. Figure S7. EV@RCA generated from varying reaction time. ..8
8. Figure S8. Optimization of EV@RCA reaction………………………………………………................8
9. Figure S9. Fluorescence intensity of EV@RCA produced under series concentrations of EV.9
10. Figure S10. Measurement different concentration of EV using a commercial chip………...……….…..9
11. Figure S11. Long-term stability of EV@RCA .11
12. Figure S12. Stability of EV@RCA under UV source.12
13. Figure S13. DPPSE assay for detecting EV in MCF-7 cell medium supernatants 13
14. Figure S14. Characterization of RCA_CD63+EpCAM+MUC1_ products.14
15. Figure S15. Results of molecular crowding test…....................................................................................15
16. Figure S16. Identification the super-bright spots signal coming from EV@RCA..................................16
17. Figure S17. NTA analysis size and concentration of EV from different cell lines .17
18. Figure S18. Analysis of EV captured on biochip using plasma with different dilution times .18
19. Figure S19. NTA analysis size and concentration of particles in plasmas from HD .20
20. Figure S20. NTA analysis size and concentration of particles in plasmas from BC .21
21. Figure S21. NTA analysis size and concentration of particles in plasmas from LAC and LSC 22
22. Figure S22. NTA analysis size and concentration of particles in plasmas from AML, B-ALL and T-ALL 23
23. Figure S23. Characterization of plasma EV 24
24. Figure S24. Analysis of the interference from miRNA in plasma for DPPIE assay……………………..24
25. Figure S25. DPPIE analysis of the specificity of DPPIE for EV detection in plasma………………….25
26. Figure S26. Linear detection of the tumor-derived EV in plasma sample………………….....………..25
27. Figure S27. Representative fluorescent images of EV@RCA of plasma samples from HD26
28. Figure S28. Representative fluorescent images of EV@RCA of plasma samples from BC27
29. Figure S29. Representative fluorescent images of EV@RCA of plasma sample from LAC and LSC.28
30. Figure S30. Representative fluorescent images of EV@RCA of plasma sample from AML, B-ALL and T-ALL.29
31. Figure S31. 3D scatter plots of individual-EV information from six randomly clinical samples.30
32. Figure S32. t-SNE analysis of 500 individual EV in HD14 and BC2 samples………………………….30
33. Figure S33. Univariate logistic regression model was used to evaluate the methods…………………...31
34. Table S1. Algorithm: Simple version of t-Distributed Stochastic Neighbor Embedding…………….....31
35. Table S2. Size distribution of EV detected by NTA in scatter and fluorescence modes……............….32
36. Table S3. Results of size and concentration distribution of different EV32
37. Table S4. Results of size and concentration distribution of particles in plasmas from HD33
38. Table S5. Results of size and concentration distribution of particles in plasmas from BC34
39. Table S6. Results of size and concentration distribution of particles in plasmas from LAC and LSC35
40. Table S7. Results of size and concentration distribution of particles in plasmas from AML, B-ALL and T-ALL36
41. Table S8. Oligonucleotide sequences used in this work.37
42. Table S9. Summary of the clinical plasma samples……………………………………………….……38
43. Table S10. Information of plasma sample from cancer patients…………………………………..….....39
44. Table S11. Information of plasma sample from healthy donors.41

**Results**

**
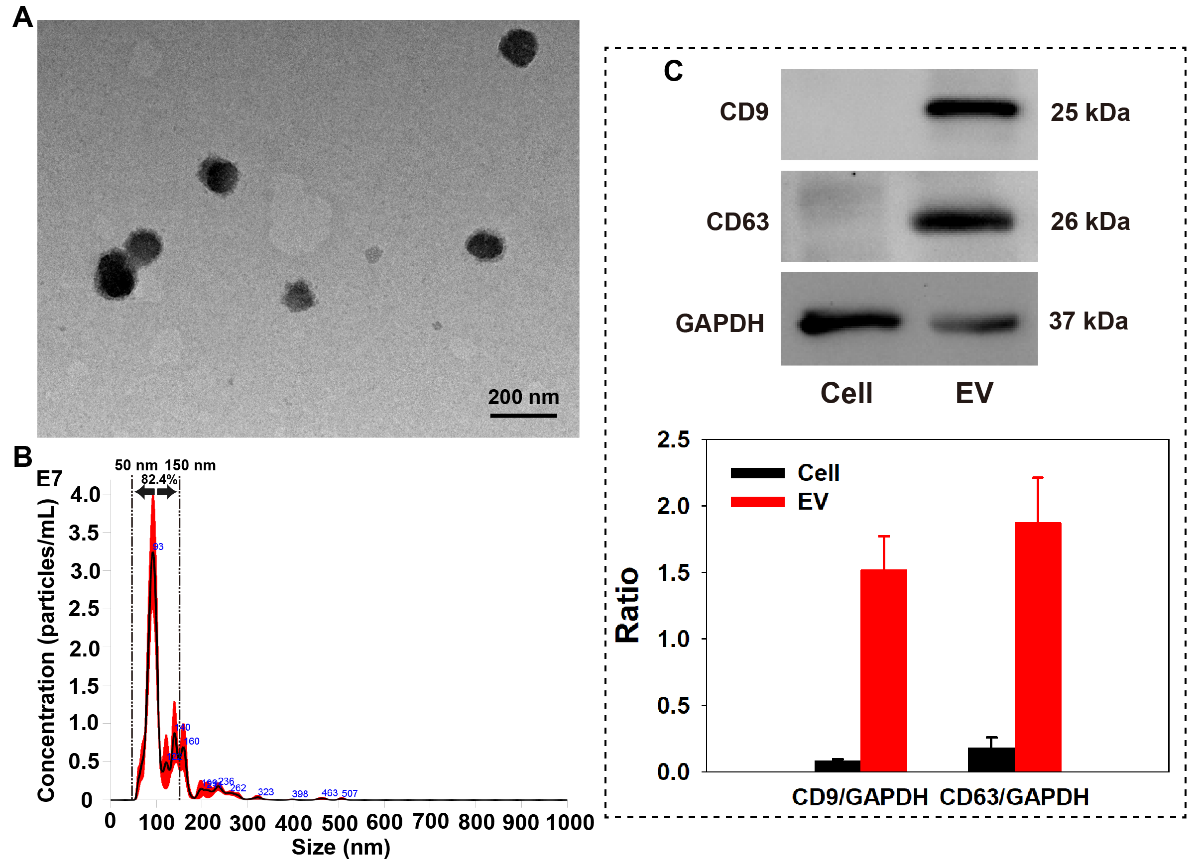
**

**Figure S1.** Characterization of EV. (A) Representative TEM image of MCF-7 EV. (B) NTA analysis of MCF-7 EV, the size of 50-150 nm accounted for 82.4%, and red band depicts three repetitive experiments. (C) Western Blot and semi-quantitative analysis the expression of CD63 and CD9 in MCF-7 EV and its parent cells. Each lane was loaded with 40 μg of total proteins. Error bars show the standard deviation of three replicate experiments.


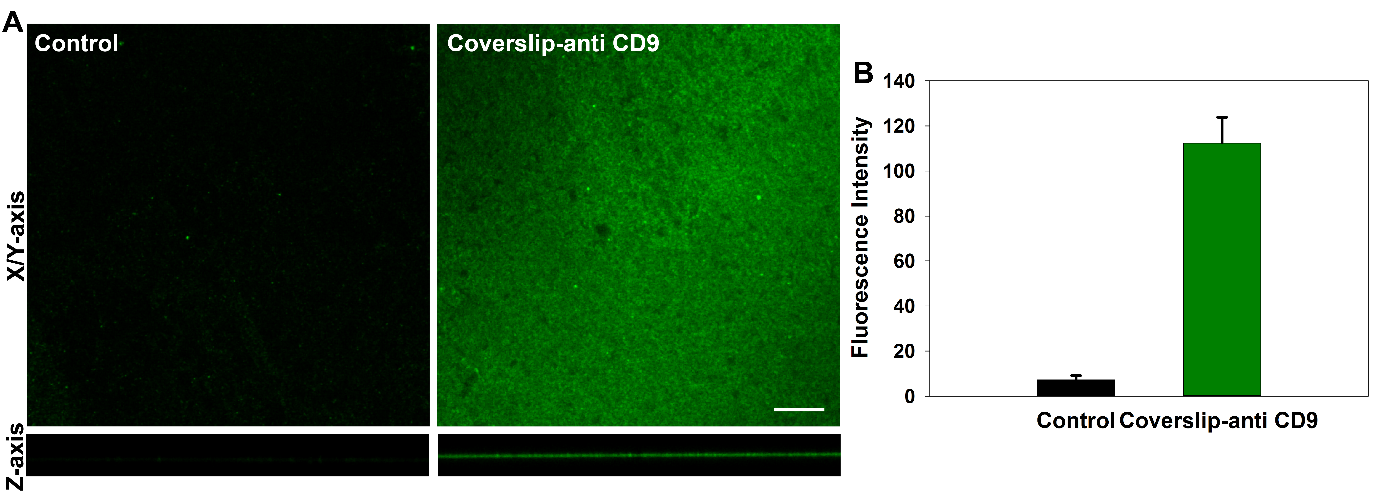


**Figure S2.** Identification the surface of biochip coated with anti-CD9. (A) CLSM images of the bared and anti-CD9 coated coverslip substrate. Scale bar: 7.5 μm. (B) Quantitative analysis of fluorescence intensity of mouse anti-Rabbit IgM/Alexa Fluor 488 conjugated without (column “control”) or with (column “Coverslip-anti CD9”) anti-CD9 antibody functionalized biochip.

As can be seen from Table S2, the size of EV was basically consistent in scatter and fluorescence modes, mainly ranging from 50 nm to 240 nm with a peak size around 150 nm. Moreover, the total EV count was 1.6 × 10^8^ particles/mL, whereas the labeled EV count was 1.3 × 10^8^ particles/mL (Figure S3). According to the results, CD9^+^ EV approximately accounting for 81.25% of all the EV and the size range showed no obvious difference.


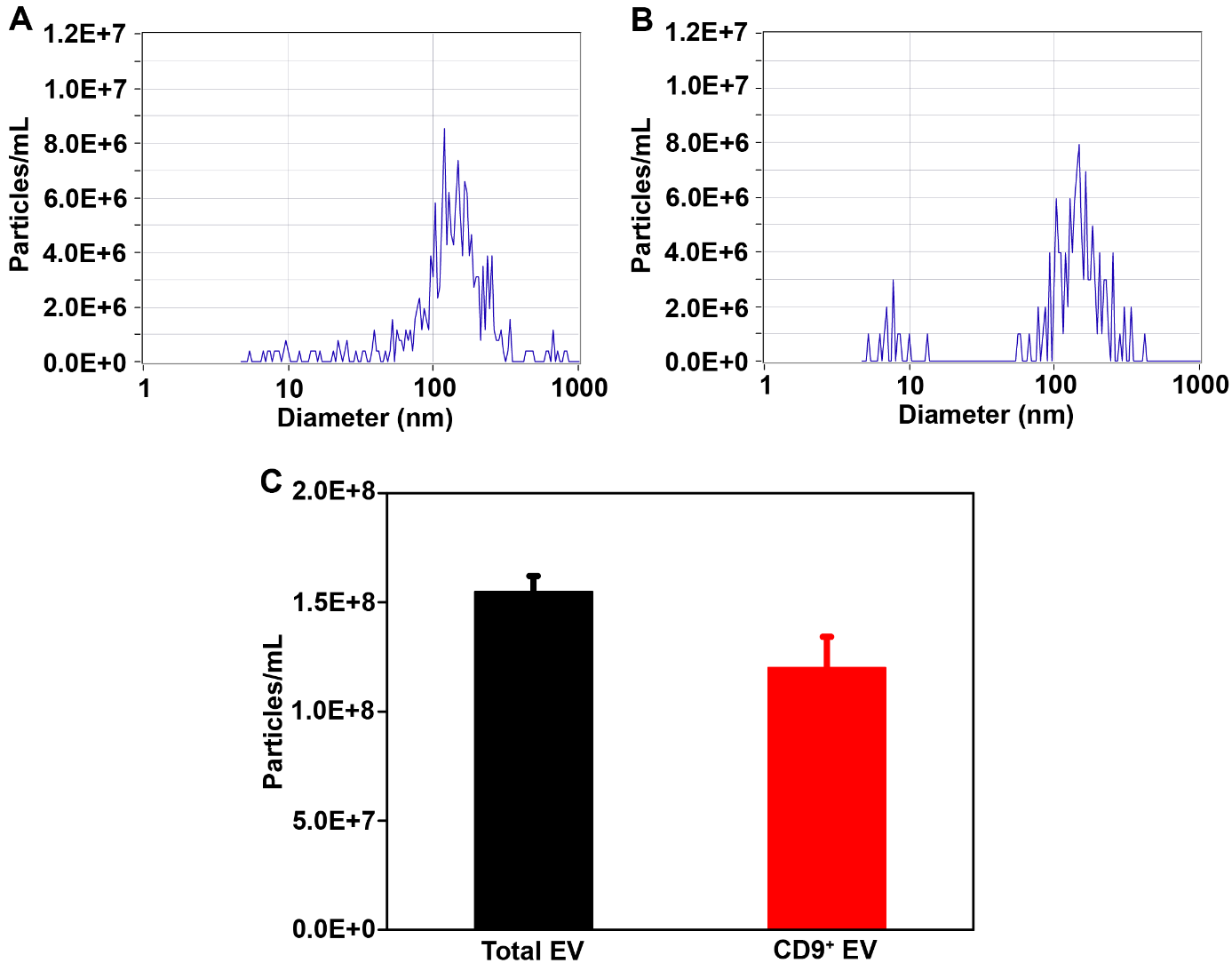


**Figure S3.** NTA analysis of EV in the scatter and fluorescence mode. (A) Size distribution of EV detected by NTA in the scatter mode. (B) Size distribution of EV detected by NTA in the fluorescence mode after labelling with rabbit anti-CD9/AF488 conjugated antibody. (C) The concentration of EV detected by NTA in scatter (total EV) and fluorescence (CD9^+^ EV) modes (*P* > 0.05) .


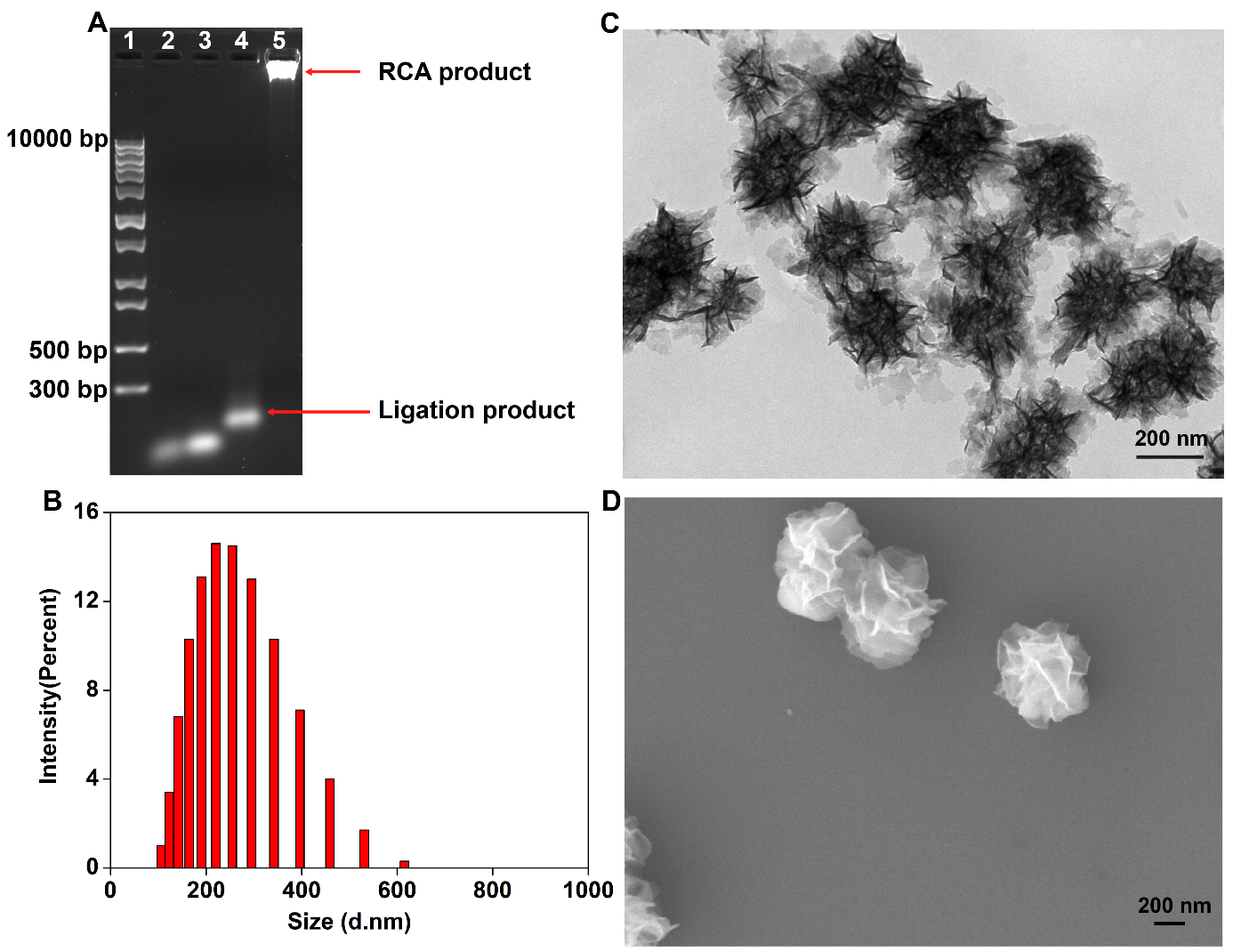


**Figure S4.** Characterization of RCA products. (A) Agarose gel (1%) electrophoresis image illustrating the elongation of DNA through RCA, lane 1-5: DNA marker, padlock probe**,** ligation template, ligation products (padlock probe + ligation template) and RCA products. (B) The size of the RCA amplicons analyzed by DLS. (C) TEM image showed that RCA amplicons were uniform monodisperse particle ranging from 300 to 400 nm with petal-like structure. (D) SEM image further verified similar “nanoflower” structure of RCA amplicons.


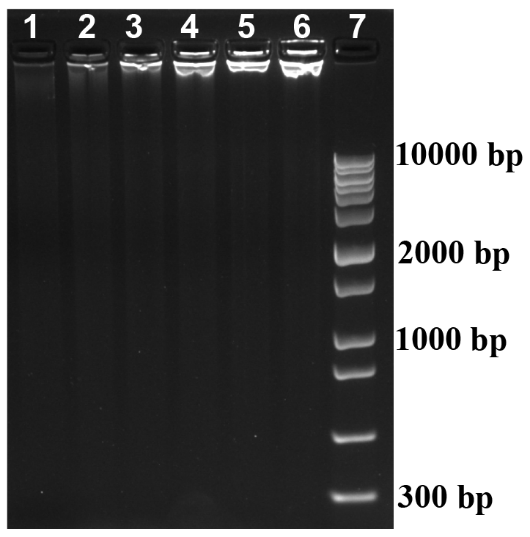


**Figure S5.** Agarose gel (1%) electrophoresis analysis of RCA products at varying reaction time. Lane 1-7: 5, 10, 20, 30, 45, 60 min and DNA marker.

**
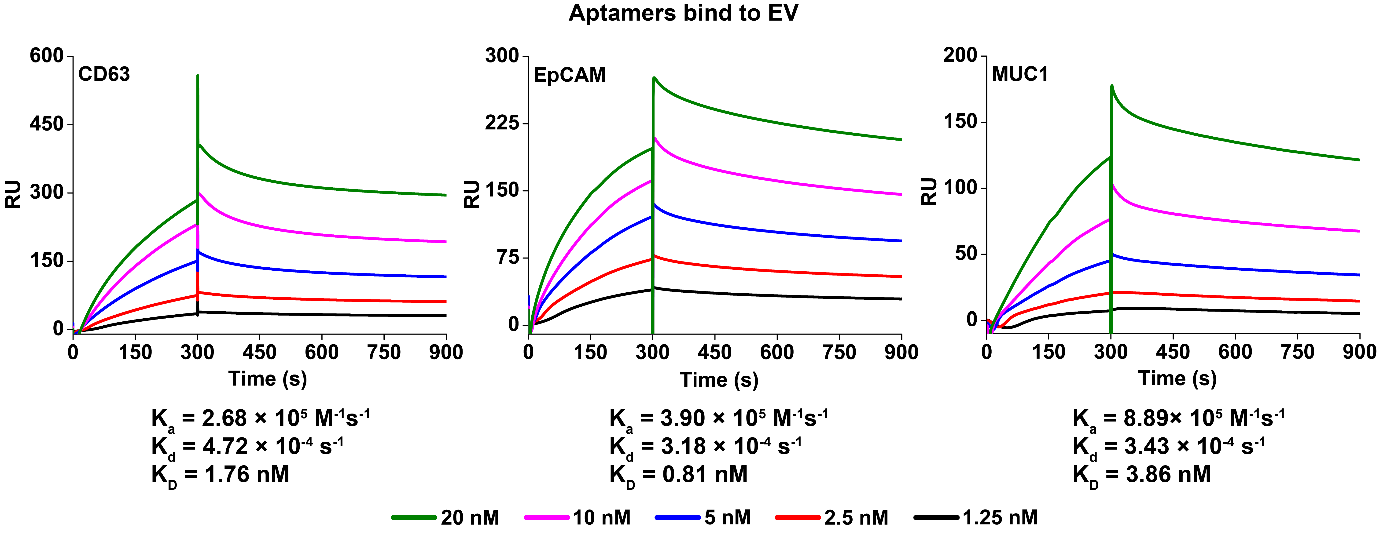
**

**Figure S6.** Kinetic analysis of EV-aptamer interaction. Association and dissociation curves of EV (concentration range from 20 nM to 1.25 nM) with biotinylated aptamer_CD63_, aptamer_EpCAM_ and aptamer_MUC1_ immobilized on streptavidin sensor chips. RU (one RU is equivalent to one picogram per square millimeter on the sensor surface).


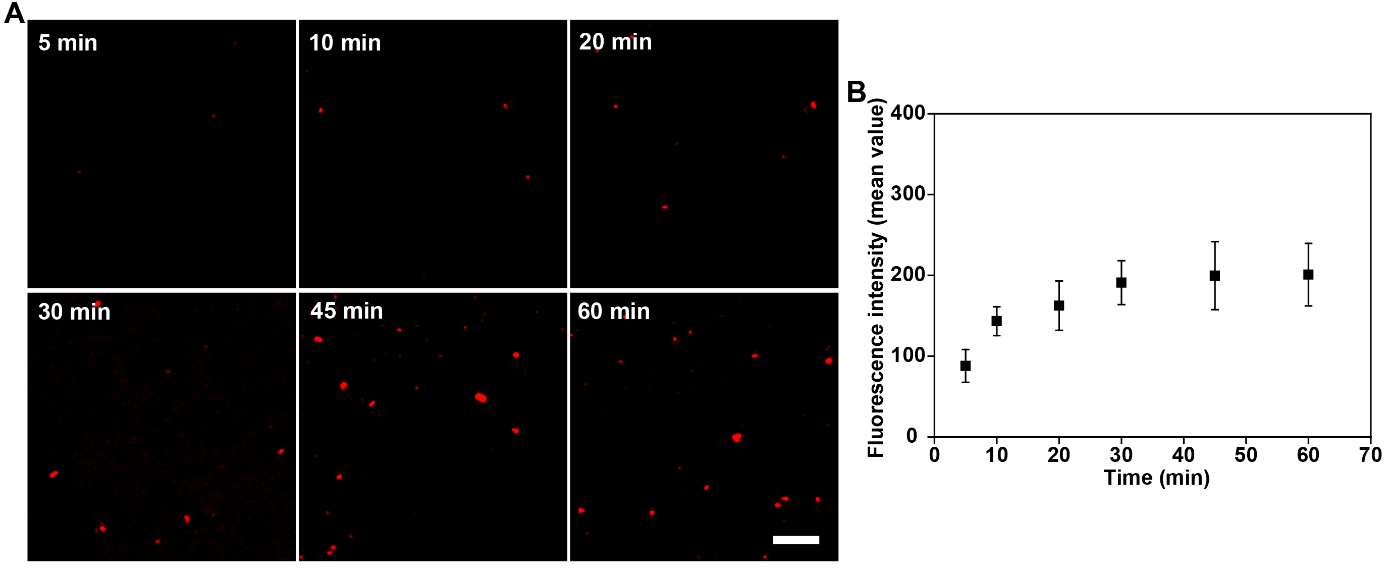


**Figure S7.** EV@RCA generated from varying reaction time. (A) Representative CLSM images of EV@RCA generated from varying reaction time. Scale bar: 7.5 μm. (B) Fluorescence intensity of EV@RCA vs. reaction time corresponding to A.


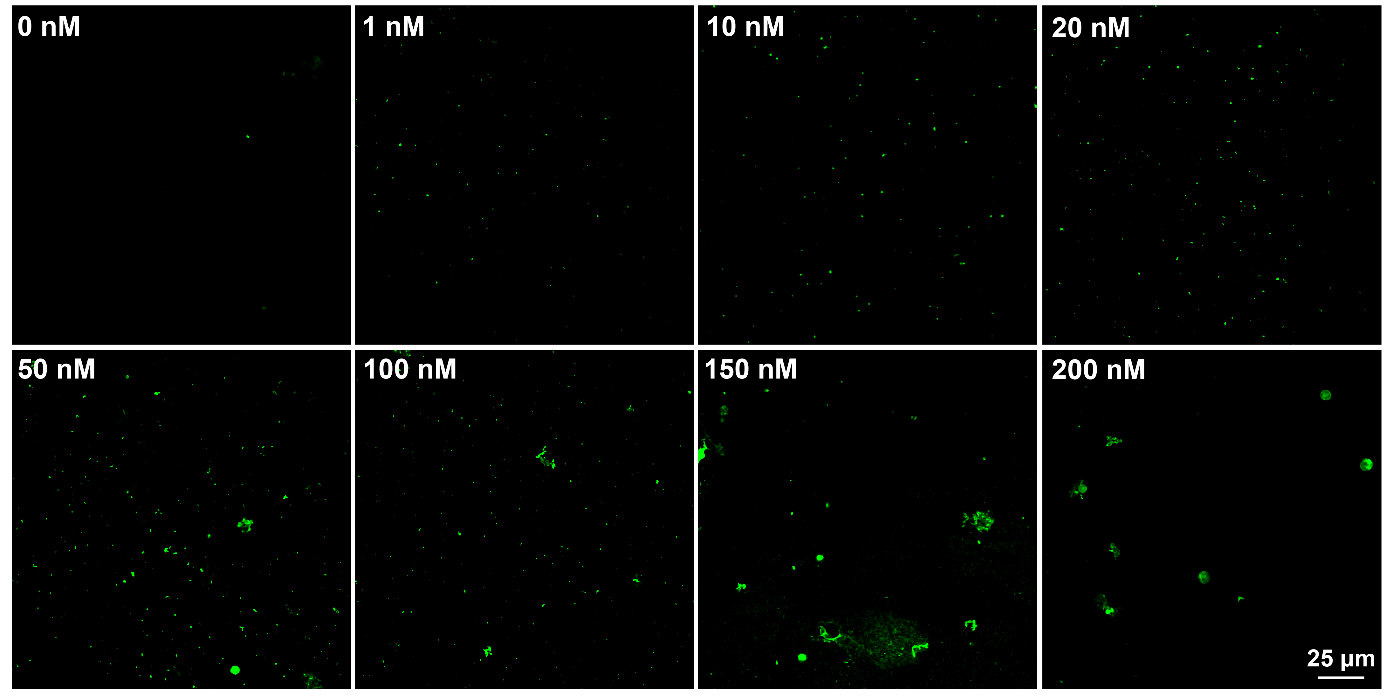


**Figure S8.** Optimization of EV@RCA reaction. DPPSE assay test EV@RCA using the various concentrations of ligation template. Scale bar: 25 μm.

**
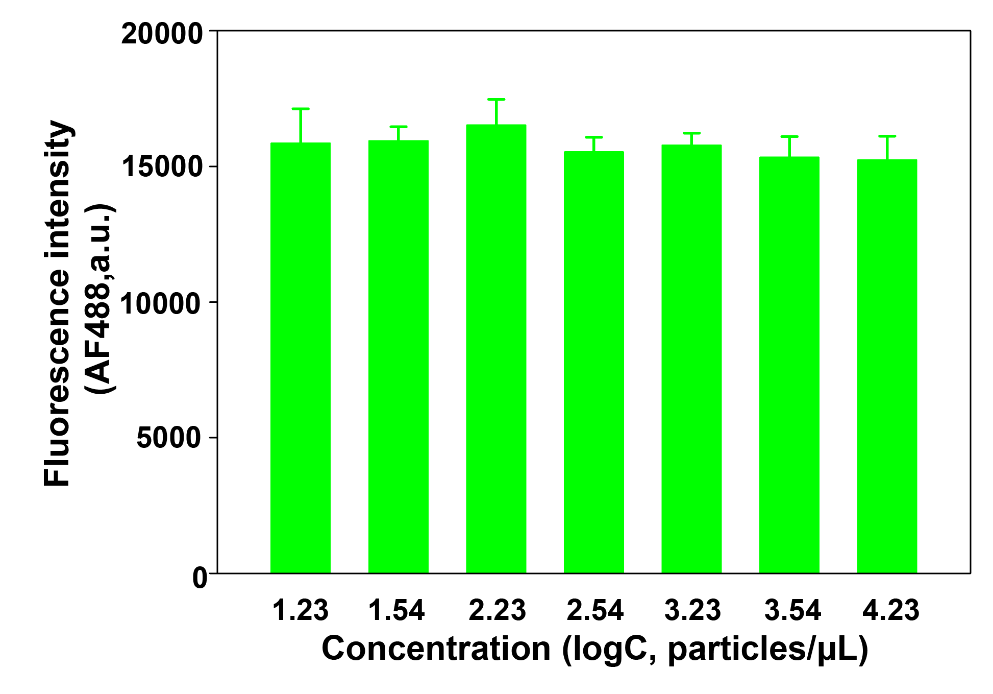
**

**Figure S9.** Fluorescence intensity of EV@RCA produced under series concentrations of EV.


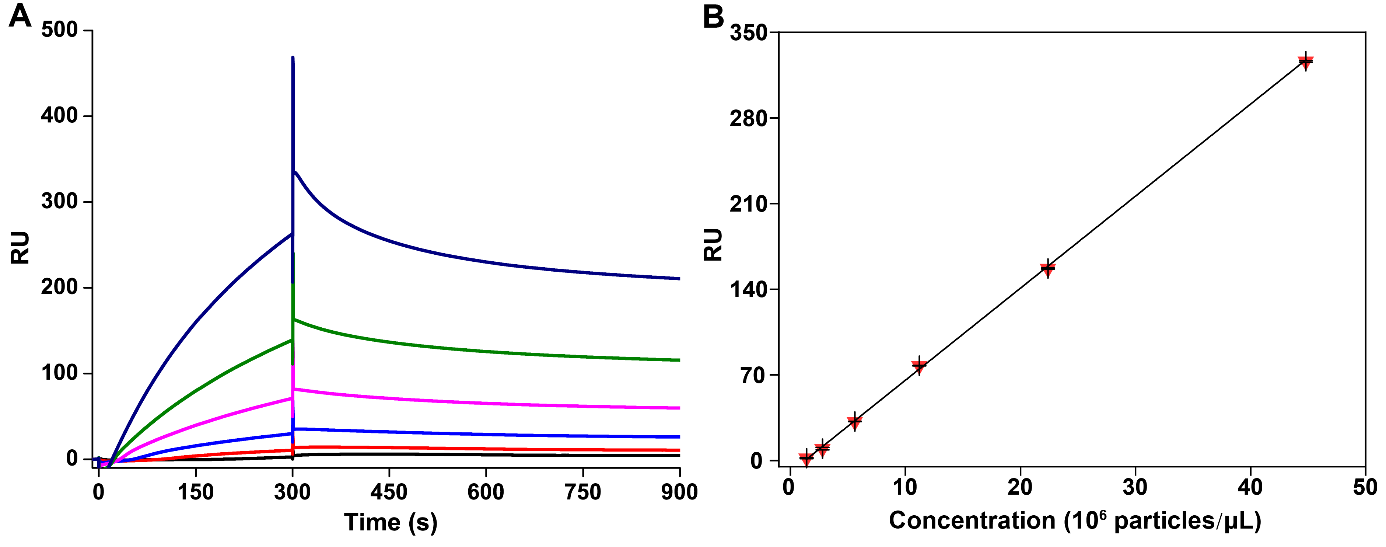


**Figure S10.** Measurement different concentration of EV using a commercial chip. (A) Blank-subtracted SPR sensor grams for measuring EV at decreasing concentrations (44.8, 22.4, 11.2, 5.6, 2.8, 1.4×10^6^ particles/μL, from top to bottom). (B) The linear relationship between RU and concentration of EV, and the correlation equation was$Y=7.52 X-9.50$ with a correlation coefficient of R^2^ = 0.99 (n = 3). RU (one RU is equivalent to one picogram per square millimeter on the sensor surface). Error bars represent the standard deviation of three replicate experiments.

To investigate the stability of EV@RCA in clinical sample, DPPSE assay was used to profile individual EV from plasma. [A](javascript:;) large number [of](javascript:;) monodispersed white, yellow, green, red and blue spots were observed in the merged picture of CLSM images (Figure S11A), which demonstrated that DPPSE assay could measure directly plasma EV. The above products were further imaged after storing for 7, 14 and 21 days, respectively. Fluorescence intensity distribution (Figure S11B) showed little change, and remained physically stable for 21 days at 4 ℃. The above experiment clearly revealed that the fluorochromes, which were covalently incorporated to the DNA matrix, were exceptionally stable and did not readily leak from the RCA products. Furthermore, no significant fluorescence intensity change was observed when EV@RCA suffering successive illumination for 1 h under a UV source (254 nm, 6 W) (Figure S12 A-B).


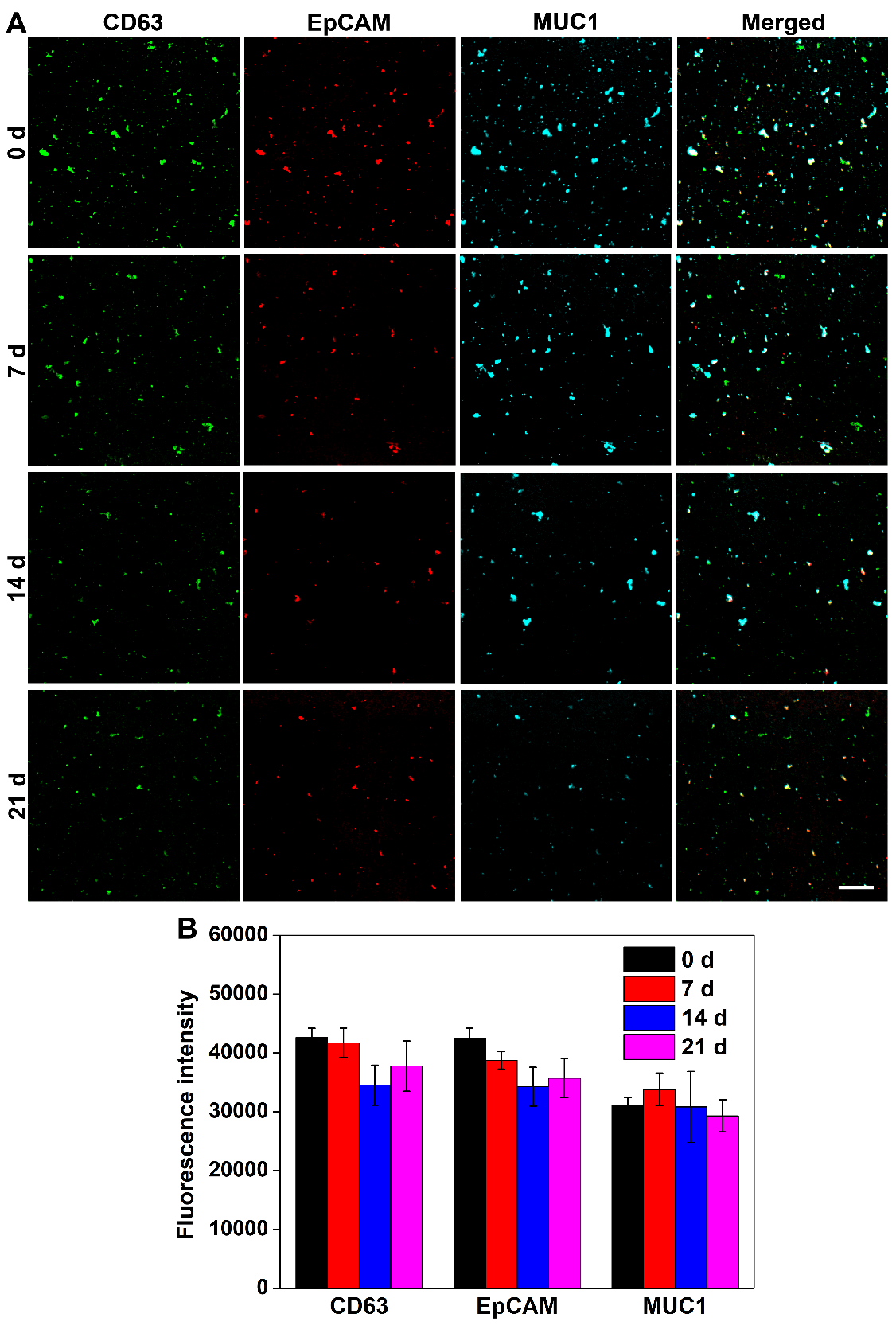


**Figure S11.** Long-term stability of EV@RCA. (A) CLSM imaging of the plasma EV@RCA after storing for 0, 7, 14 and 21 d, respectively. (B) Fluorescence intensity distribution of EV@RCA at different times. Scale bar: 10 μm.


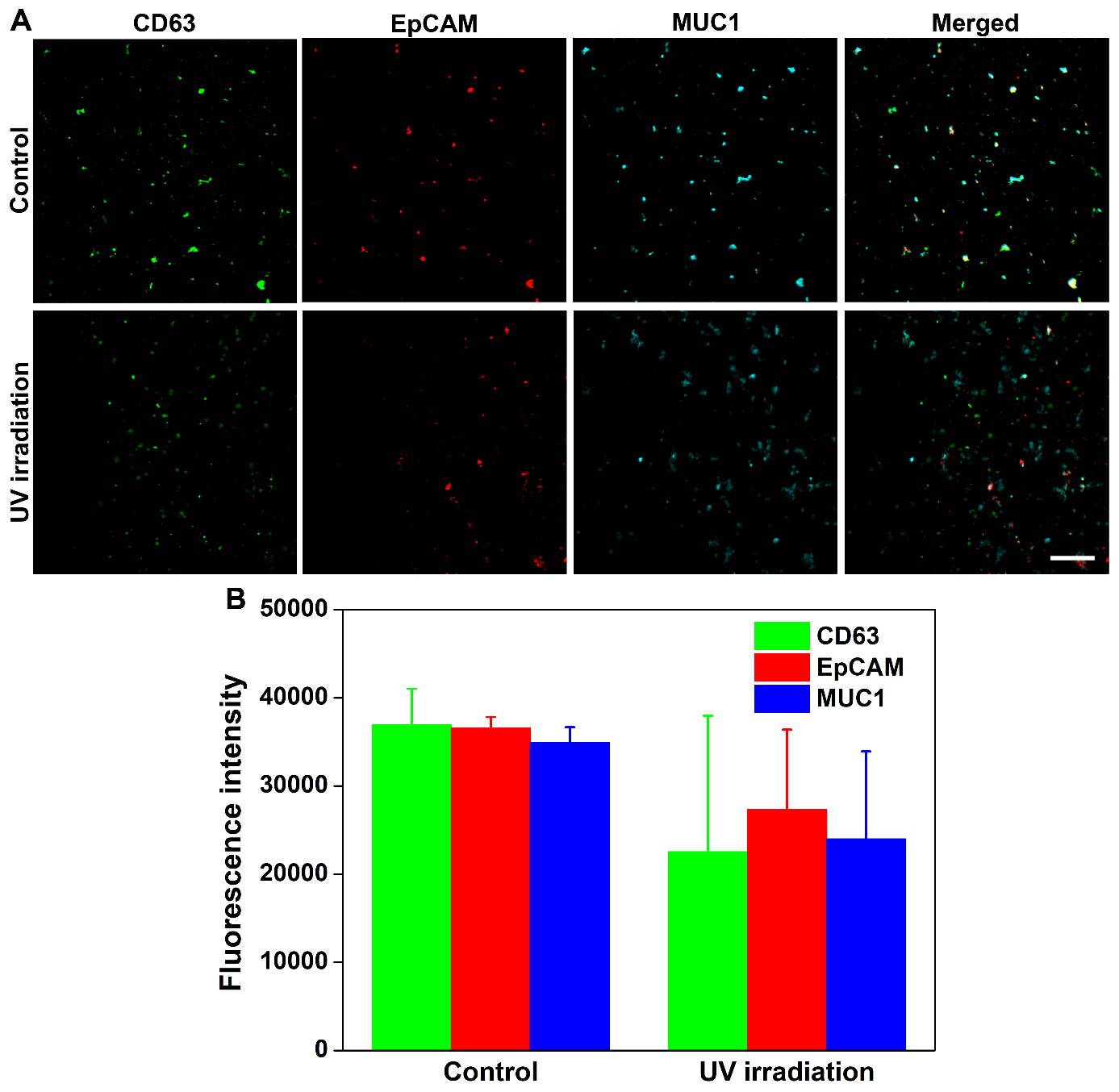


**Figure S12.** Stability of EV@RCA under UV source. (A) CLSM imaging of plasma EV@RCA without or with successive illumination with a UV source (254 nm, 6 W). (B) Quantitative analysis of fluorescence intensity of EV@RCA without or with UV source illuminating. Scale bar: 10 μm.

To further evaluate the suitability of the DPPSE for detecting EV without isolation, fresh medium supernatants from MCF-7 cell lines were analyzed. Sparse captured EV were observed in SEM images (Figure S13A), revealing that EV from cell supernatants could be captured via engineered biochip. Furthermore, a few super-bright white, yellow, green, red and blue spots were also visualized in CLSM images (Figure S13B).


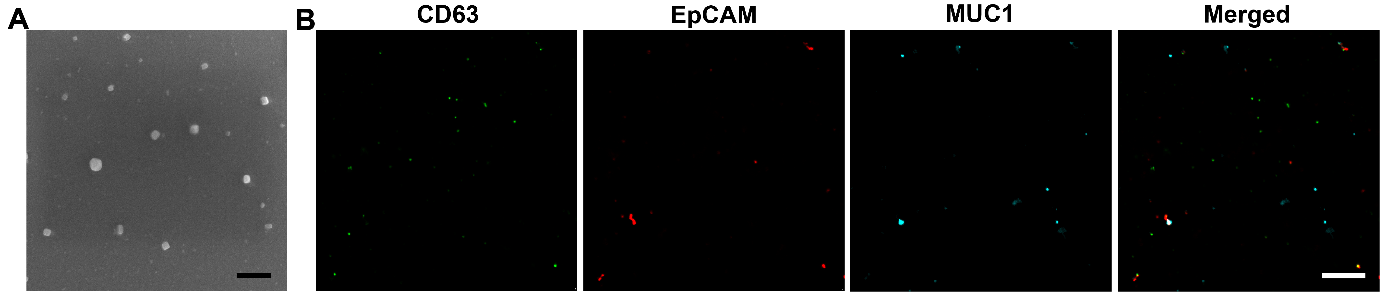


**Figure S13.** DPPSE assay for detecting EV in MCF-7 cell medium supernatants. (A) Representative SEM image of EV in MCF-7 cell medium supernatants captured by anti-CD9 functionalized biochip. Scale bar: 500 nm. (B) Representative CLSM images of EV@RCA from MCF-7 cell medium supernatants. Scale bar: 10 μm.


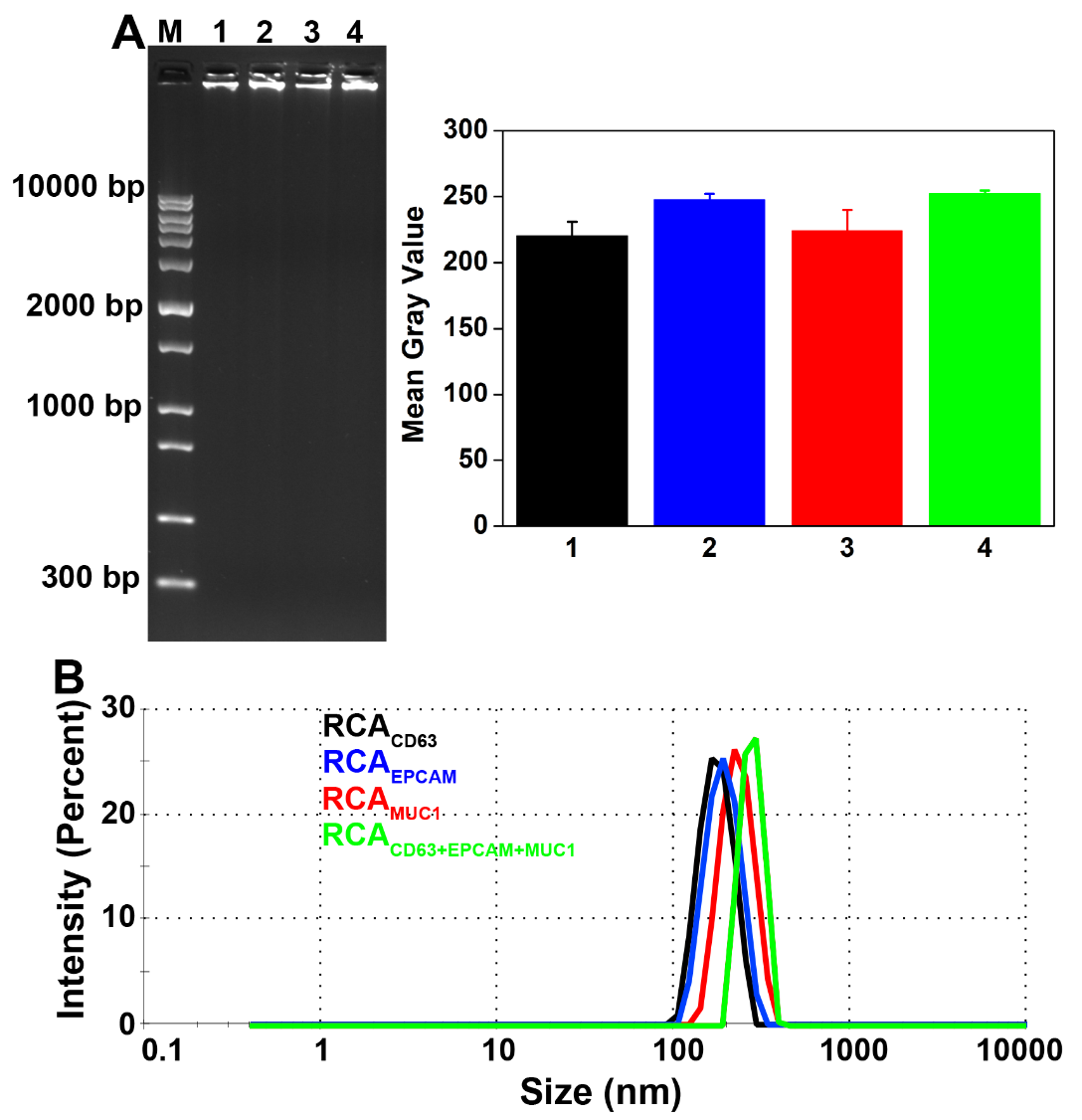


**Figure S14.** Characterization of RCA_CD63+EpCAM+MUC1_ products. (A) Agarose gel electrophoresis imaging and quantitative analysis of RCA amplicons, lane 1-4 : RCA_CD63_，RCA_EpCAM_, RCA_MUC1_ and RCA_CD63+EpCAM+MUC1_, lane M: DNA marker. (B) DLS analysis of size distribution of RCA amplicons.


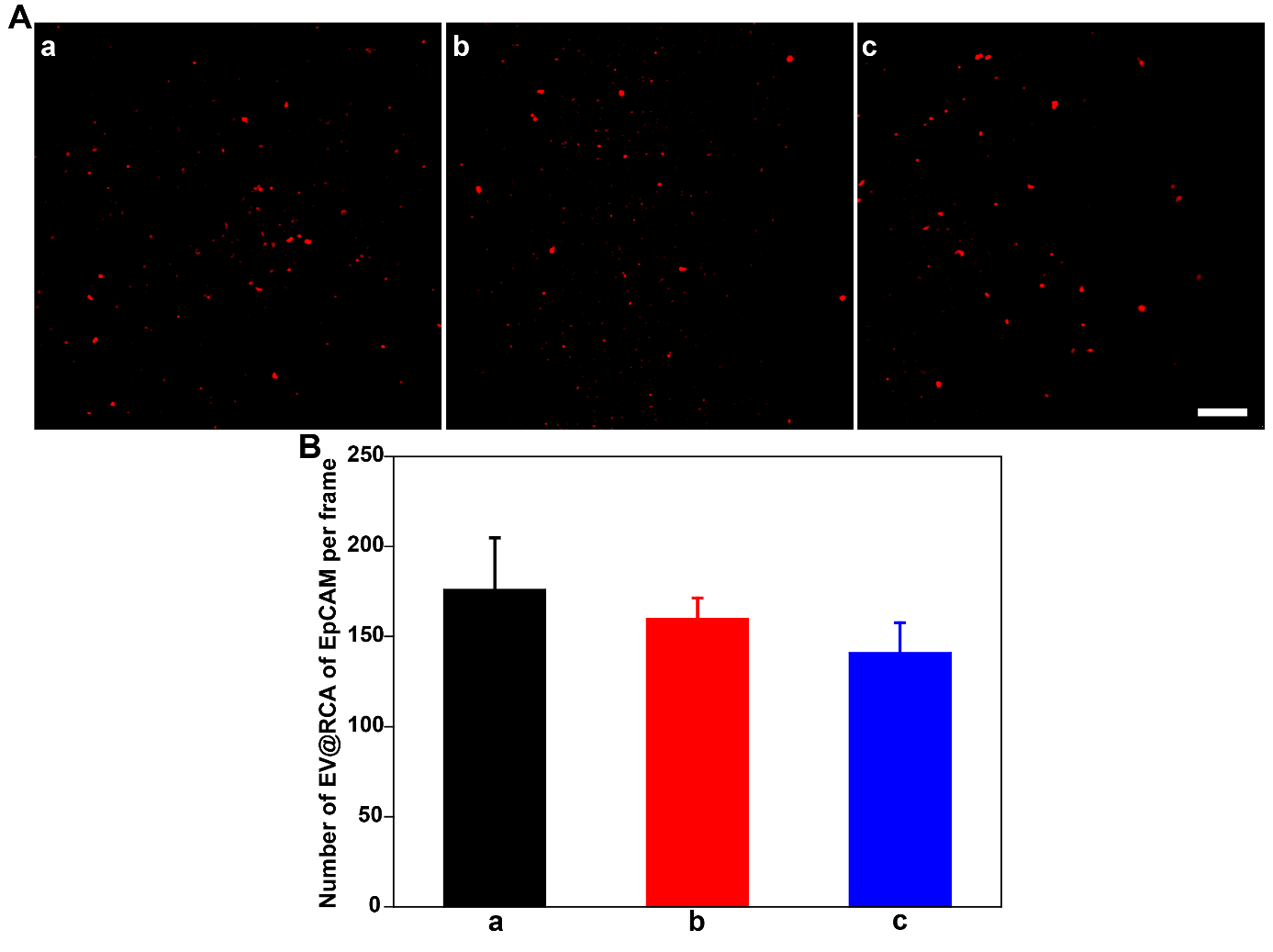


**Figure S15.** Results of molecular crowding test. (A) CLSM imaging of EV@RCA_EpCAM_ with aptamers labeled EV, respectively. (a: aptamer_EpCAM_; b: aptamer_CD63_+ aptamer_EpCAM_; c: aptamer_CD63_+ aptamer_EpCAM_+ aptamer_MUC1_). Scale bar: 7.5 μm. (B) Number of EV@RCA_EpCAM_ per frame corresponding to A (*P* > 0.05).

To ensure that the super-bright spots signal solely coming from EV@RCA, EV detection probes or absence of EV were performed as control group. No fluorescence signal distinguished from background was observed, which verified that EV did not bond with fluorophore-labeled detection probes. Additionally, biochip was blocked with block/buffer for 2 h before use when absence of EV were conducted. Compared to the experimental group, negligible bright spots were found in fluorescence images, suggesting markedly reducing the effects of non-specific adsorption (Figure S16).


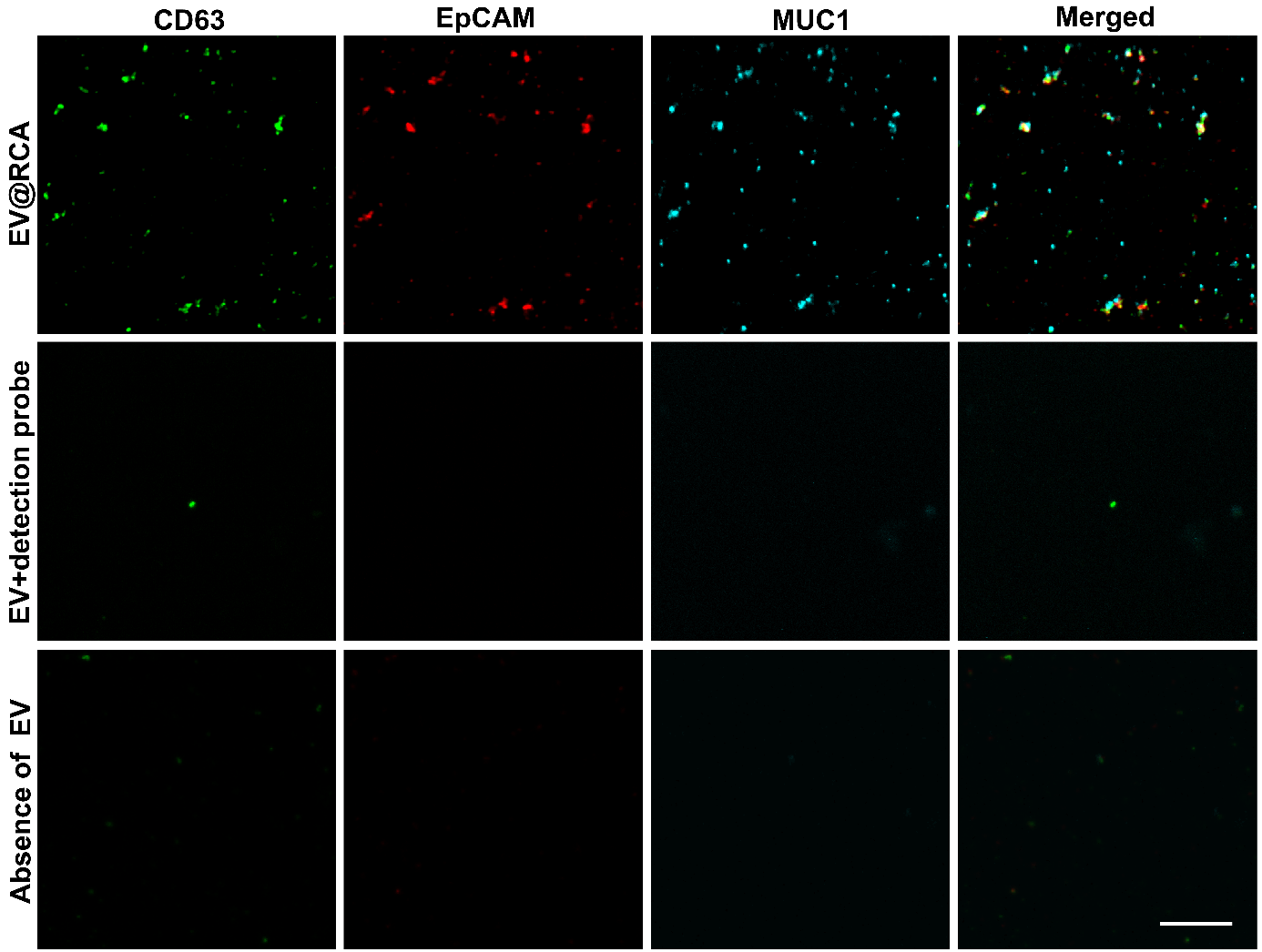


**Figure S16.** Identification the super-bright spots signal coming from EV@RCA. Scale bar: 7.5 μm.


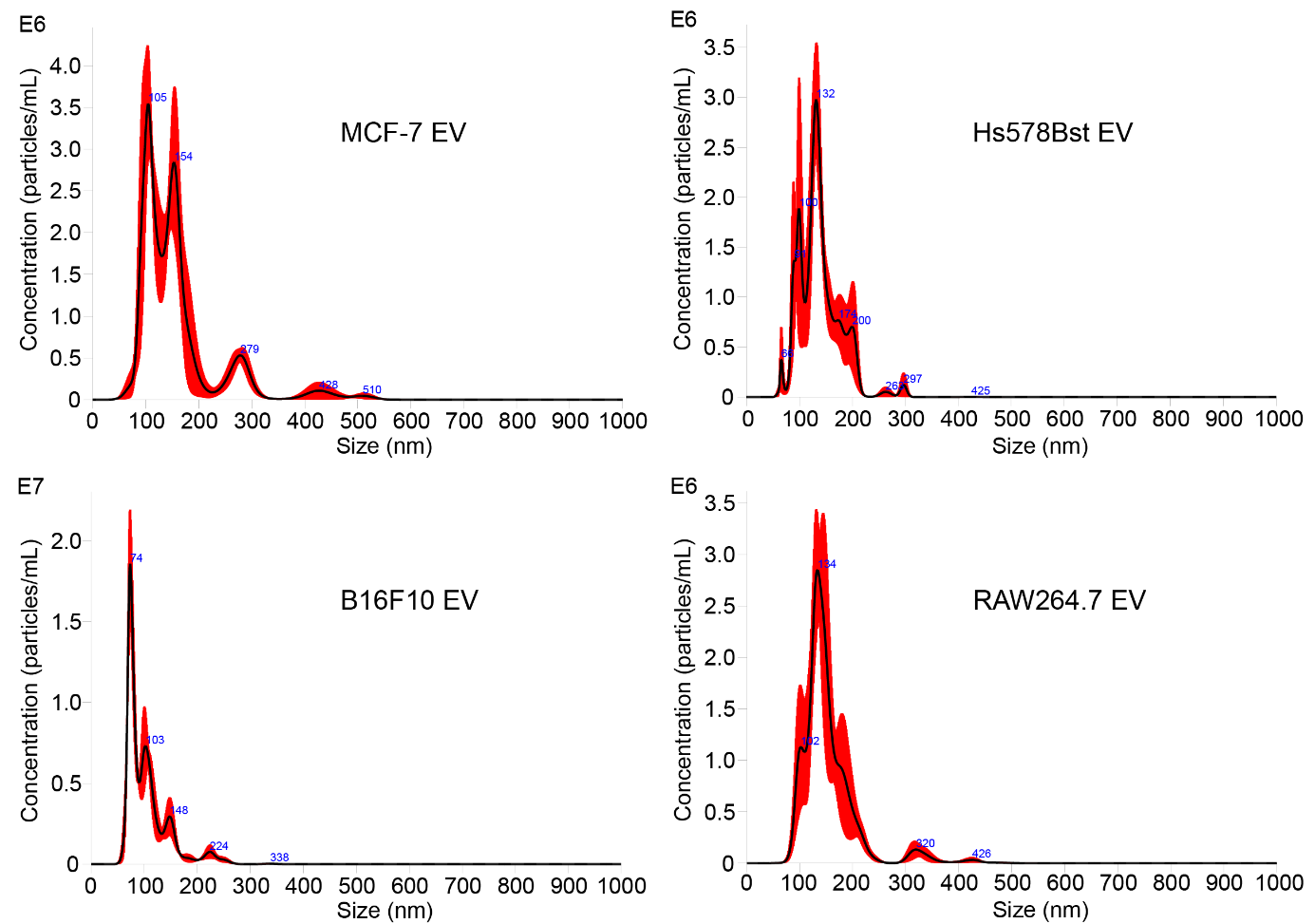


**Figure S17.** NTA analysis size and concentration of EV (dilution 200-fold with PBS) from MCF-7, Hs578Bst, B16F10 and RAW264.7 cell lines, respectively. Red band depicts three repetitive experiments.

As shown in Figure S18 A, for plasma samples with 10 times dilution to 100 times dilution, most of EV were clustered, which may affect the detection. While for plasma with 500 times dilution or 1000 times dilution, EV were mainly individually distributed on the biochip.

Afterwards, RCA reaction was performed on the chip to prepare EV@RCA_EpCAM_ for *in situ* fluorescent imaging. Similarly, when the plasma was diluted 10 or 100 times, it was mainly the large and inhomogeneous fluorescent spots observed. However, for the plasma diluted 500 or 1000 times, we can see well dispersed fluorescent signals in the images, which was suitable for individual EV analysis (Figure S18B).


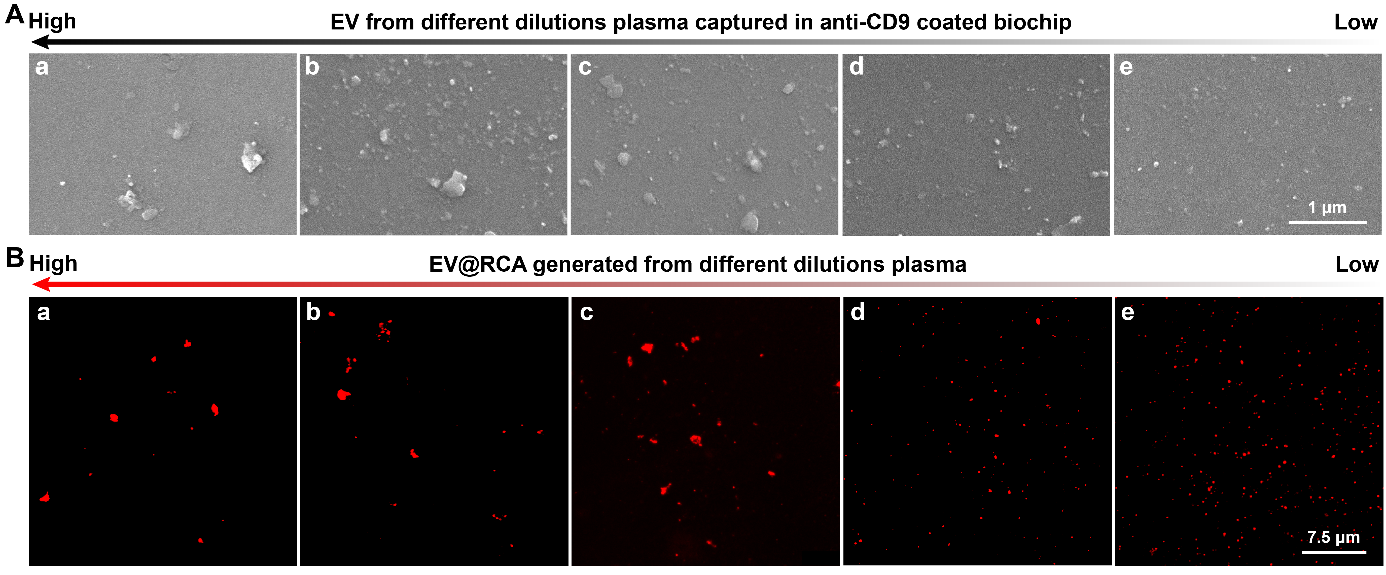


**Figure S18.** Analysis of EV captured on biochip using plasma with different dilution times. (A) SEM images of EV from different diluted plasma captured in anti-CD9 coated biochip. (B) CLSM images of EV@RCA generated from different diluted plasma. (a: Plasma; b: Plasma of 10 times dilution; c: Plasma of 100 times dilution; d: Plasma of 500 times dilution; e: Plasma of 1000 times dilution).

To further explore whether there were differences in concentration and size of plasma EV between healthy donors and cancer patients, two cohorts (healthy donors (HD, n=15) and breast cancer patients (BC, n=14)) were analyzed. Before each test, the instrument was calibrated using standard particles (100 nm polystyrene latex microspheres), and measurements were conducted follow standard protocol using a NanoSight NS300 system (Malvern Technologies, Malvern, UK) with a Blue 488 laser and sCMOS camera. All samples were measured using the same settings for head-to-head comparison. For each sample, three 30 sec videos were recorded with the following settings: temperature: 25℃, camera level: 12, number of frames: 749, viscosity: (water) 0.868–0.870 cP, slider shutter: 1232, slider gain: 219 and FPS: 25. The videos were recorded and analyzed with NanoSight NTA Software v3.2 Build 3.2.16 to measure the size and concentration of the particles. The resultant heatmap (Fig. 5C-D) was prepared on the basis of the information in Figure S19-20 and Table S4-5. Results showed that healthy donors and breast cancer patients were unable to classify into distinct clusters by size or concentration of plasma EV, and there was no significant difference among the two groups.


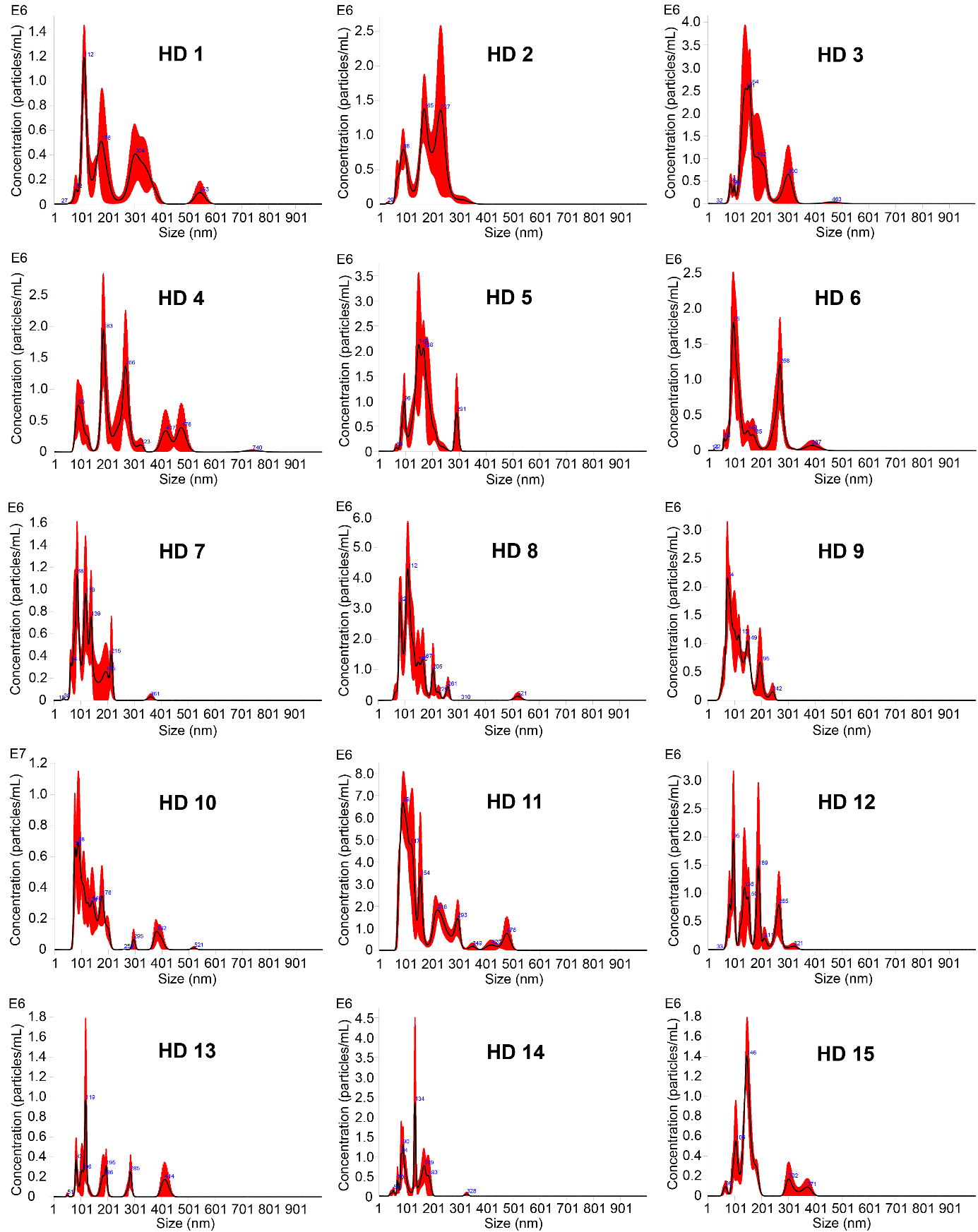


**Figure S19.** NTA analysis size and concentration of particles in plasma (dilution 1000-fold with PBS) from healthy donors (HD, n=15), respectively. Red band depicts three repetitive experiments.


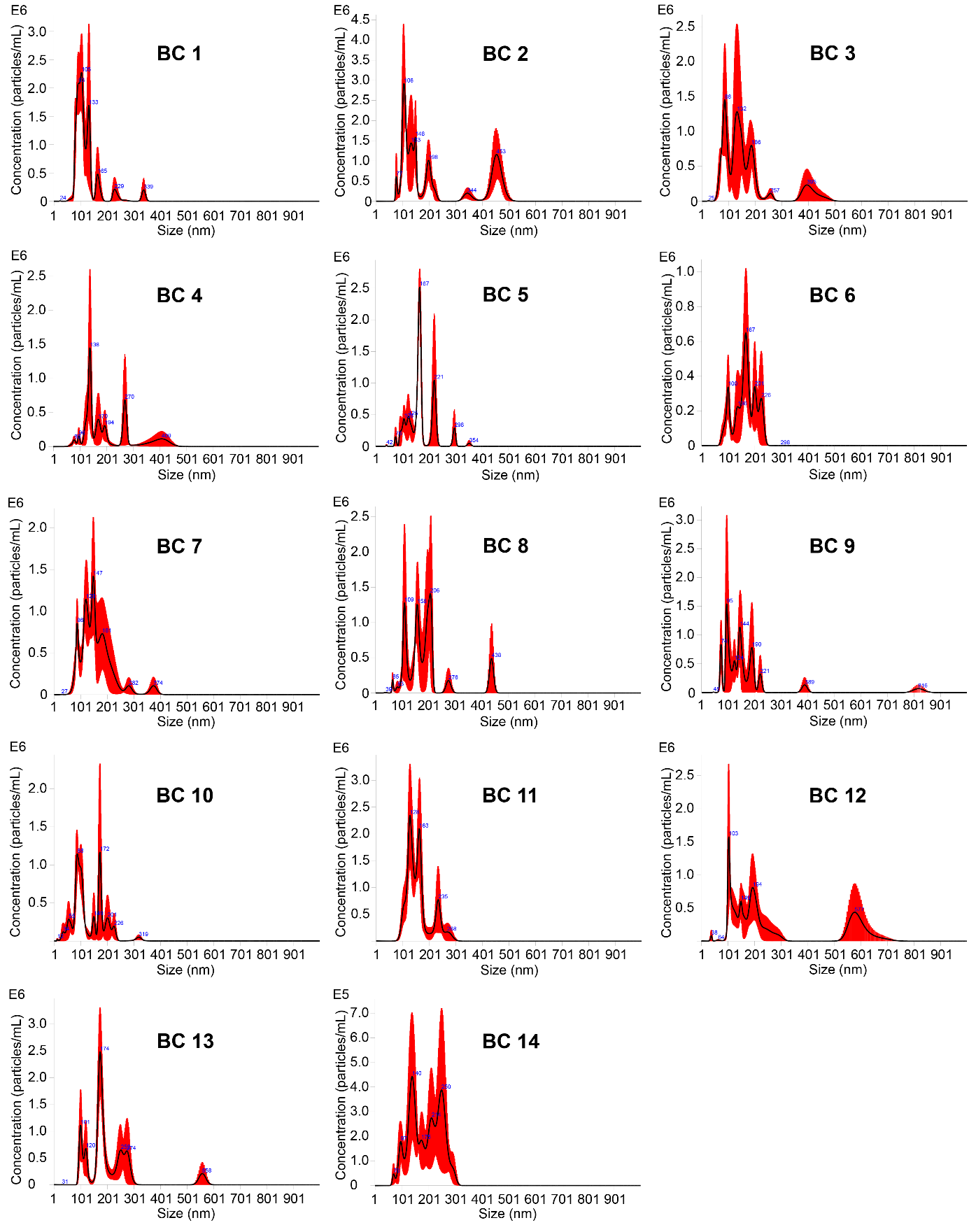


**Figure S20.** NTA analysis size and concentration of particles in plasma (dilution 1000-fold with PBS) from breast cancer (BC, n=14) patients, respectively. Red band depicts three repetitive experiments.


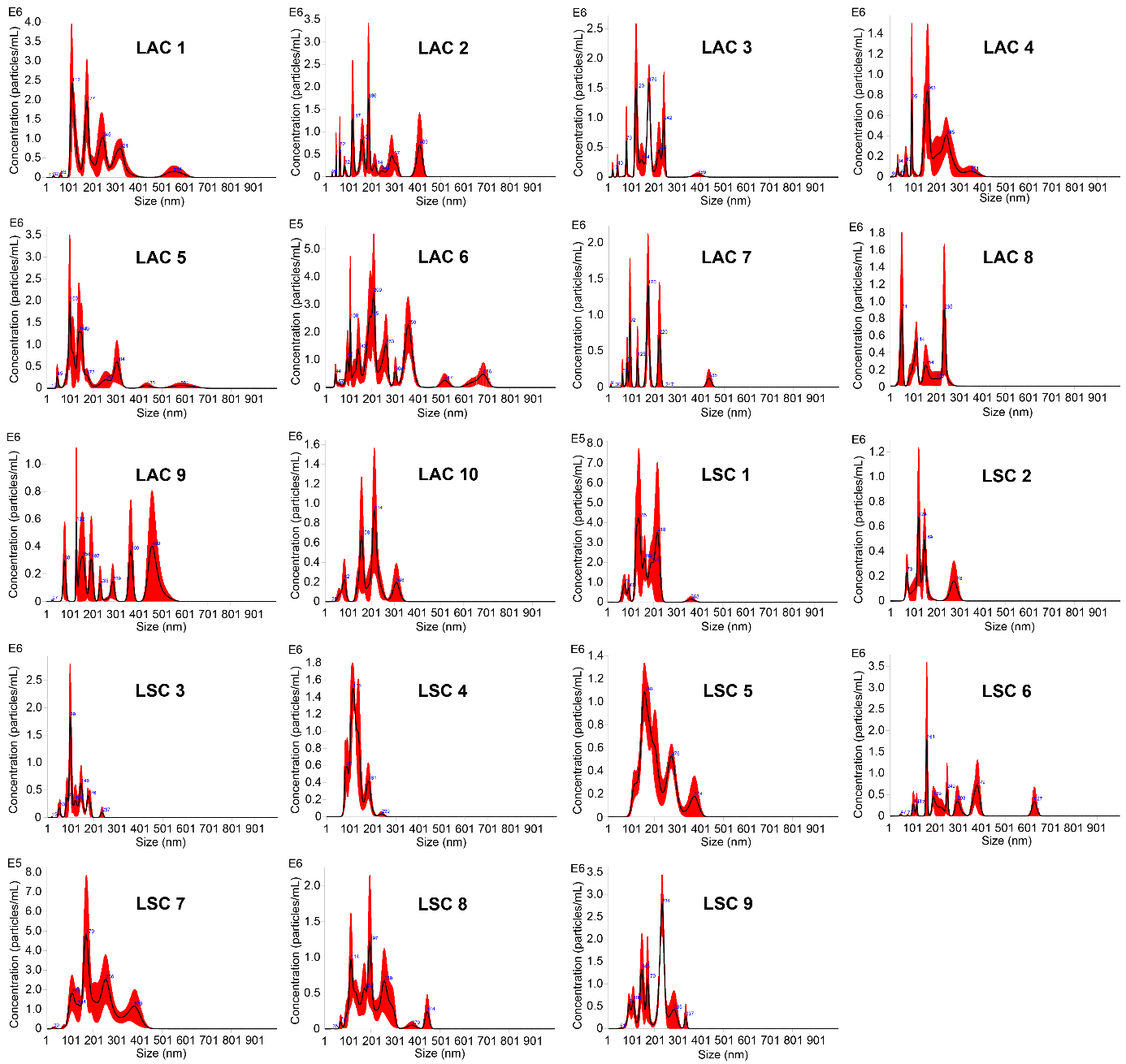


**Figure S21.** NTA analysis size and concentration of particles in plasma (dilution 1000-fold with PBS) from lung adenocarcinoma (LAC, n=10) and lung squamous carcinoma (LSC, n=9) patients, respectively. Red band depicts three repetitive experiments.


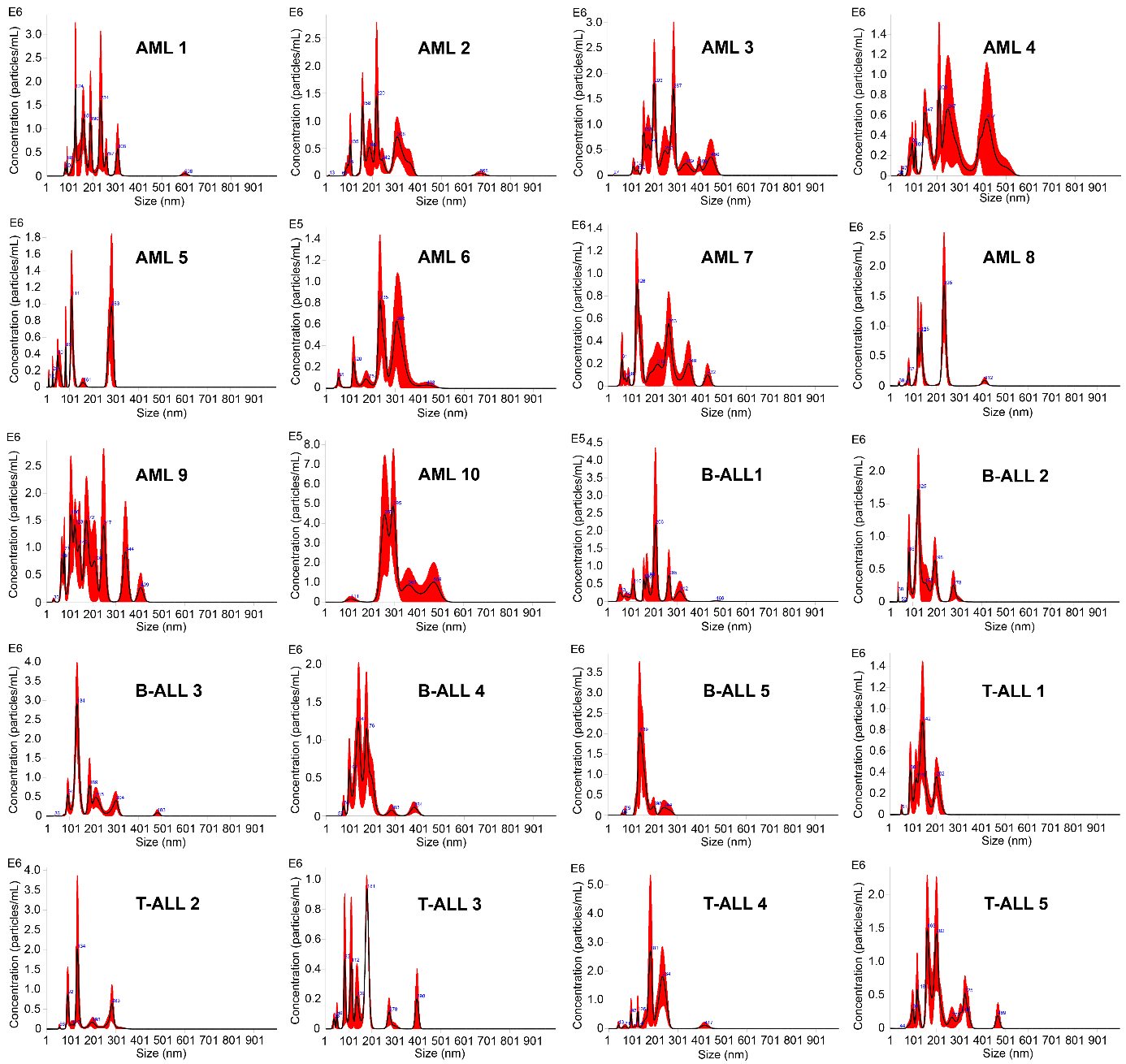


**Figure S22.** NTA analysis size and concentration of particles in plasma (dilution 1000-fold with PBS) from acute myeloid leukemia (AML, n=10), B-cell acute lymphoblastic leukemia (B-ALL, n=5) and T-cell acute lymphoblastic leukemia (T-ALL, n=5), respectively. Red band depicts three repetitive experiments.


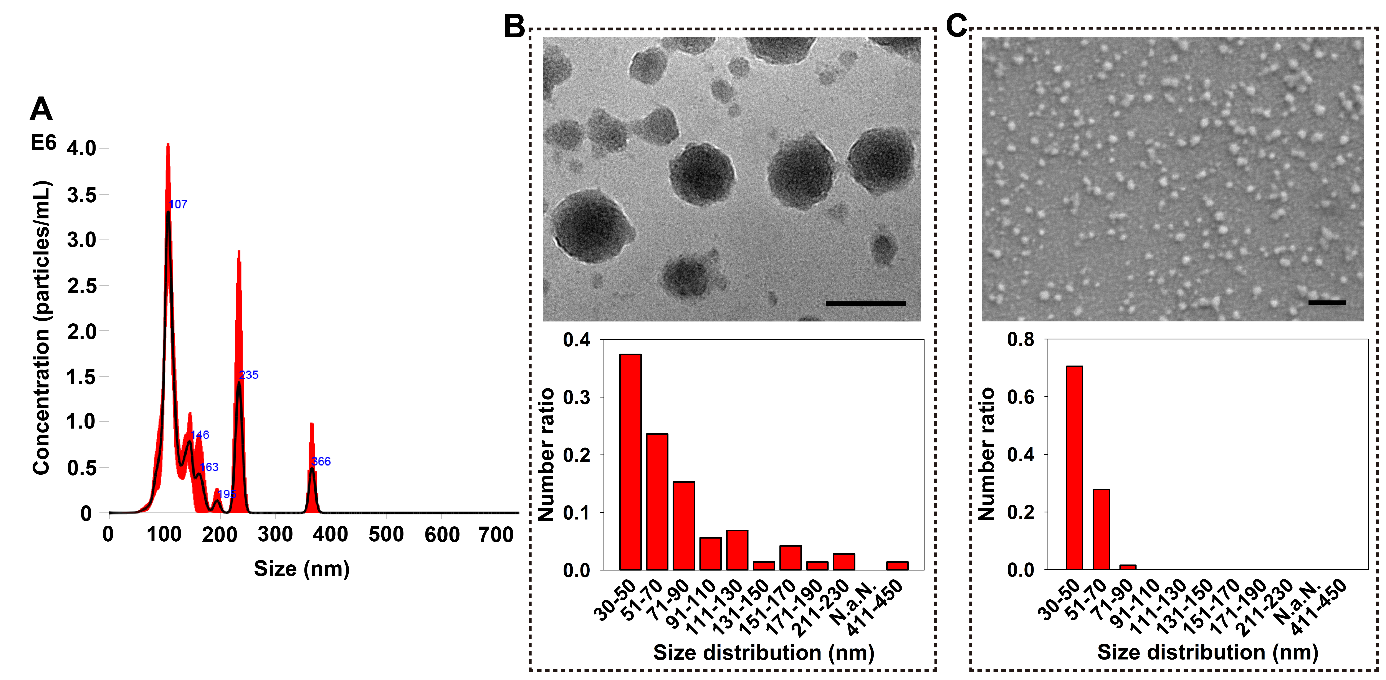


**Figure S23.** Characterization of plasma EV. (A) NTA analysis size and concentration of particles in 1000-fold diluted plasma, red band depicts three repetitive experiments. (B) Representative TEM image and particles size distribution of 1000-fold diluted plasma. Scale bar: 200 nm. (C) Representative SEM image of plasma EV captured by anti-CD9 functionalized biochip and particles size distribution. Scale bar: 200 nm.

**
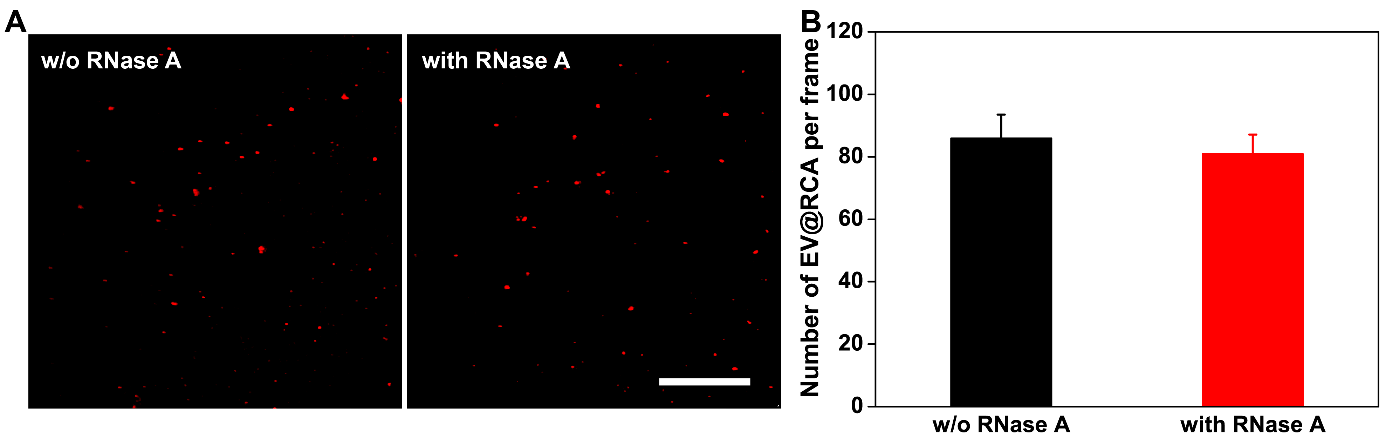
**

**Figure S24.** Analysis of the interference from miRNA in plasma for DPPIE assay. (A) CLSM images of plasma sample with or without (w/o) RNase A treatment. Scale bar : 7.5μm. (B) Number of EV@RCA per frame in plasma samples with or w/o RNase A treatment (*P* > 0.05).

**
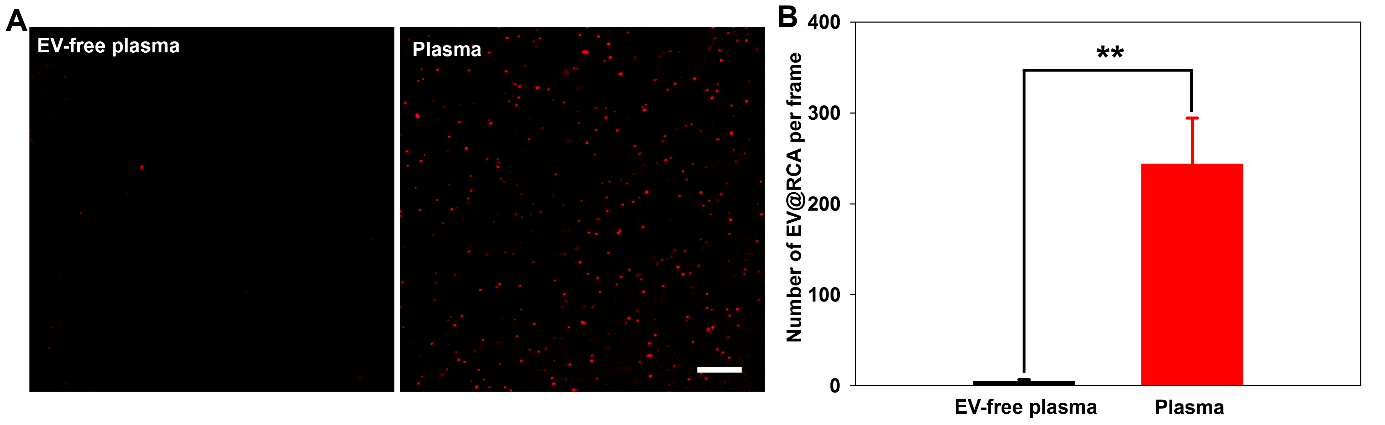
**

**Figure S25.** DPPIE analysis of the specificity of DPPIE for EV detection in plasma. (A) CLSM images of EV@RCA_EpCAM_ in EV-free plasma and regular plasma sample. Scale bar: 7.5 μm. (B) Number of EV@RCA_EpCAM_ per frame in EV-free plasma and regulate plasma sample.

**
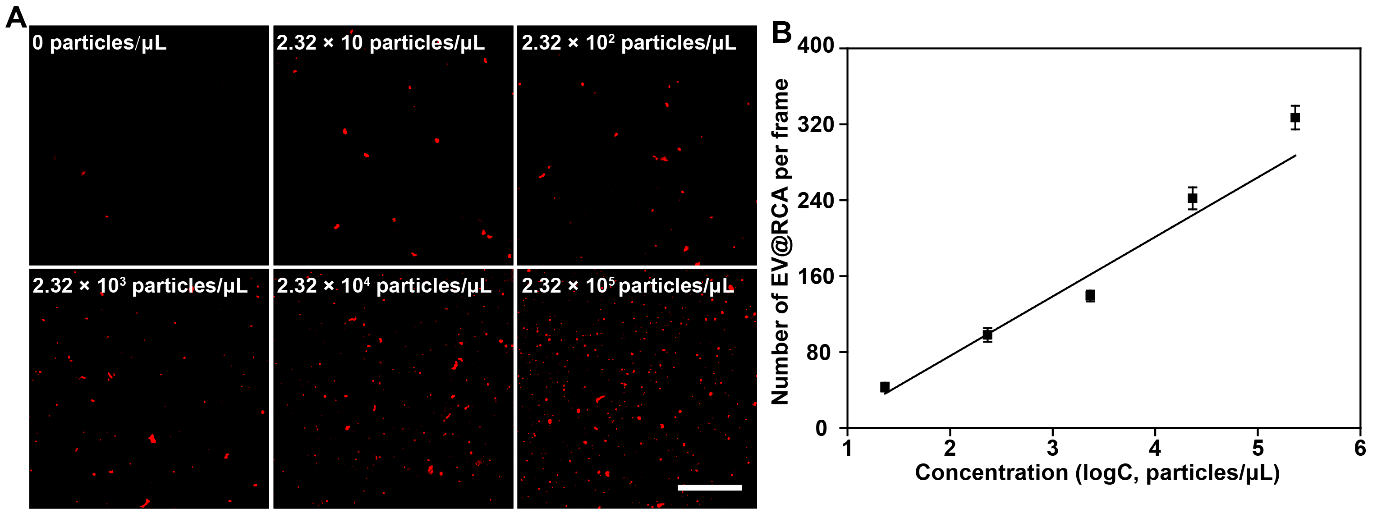
**

**Figure S26.** Linear detection of the tumor-derived EV in plasma sample. (A) CLSM images of EV@RCA detected in plasma samples with known EV concentrations. Scale bar: 3 μm. (B) Linear relationship between the number of EV@RCA per frame and log EV concentration in plasma samples.


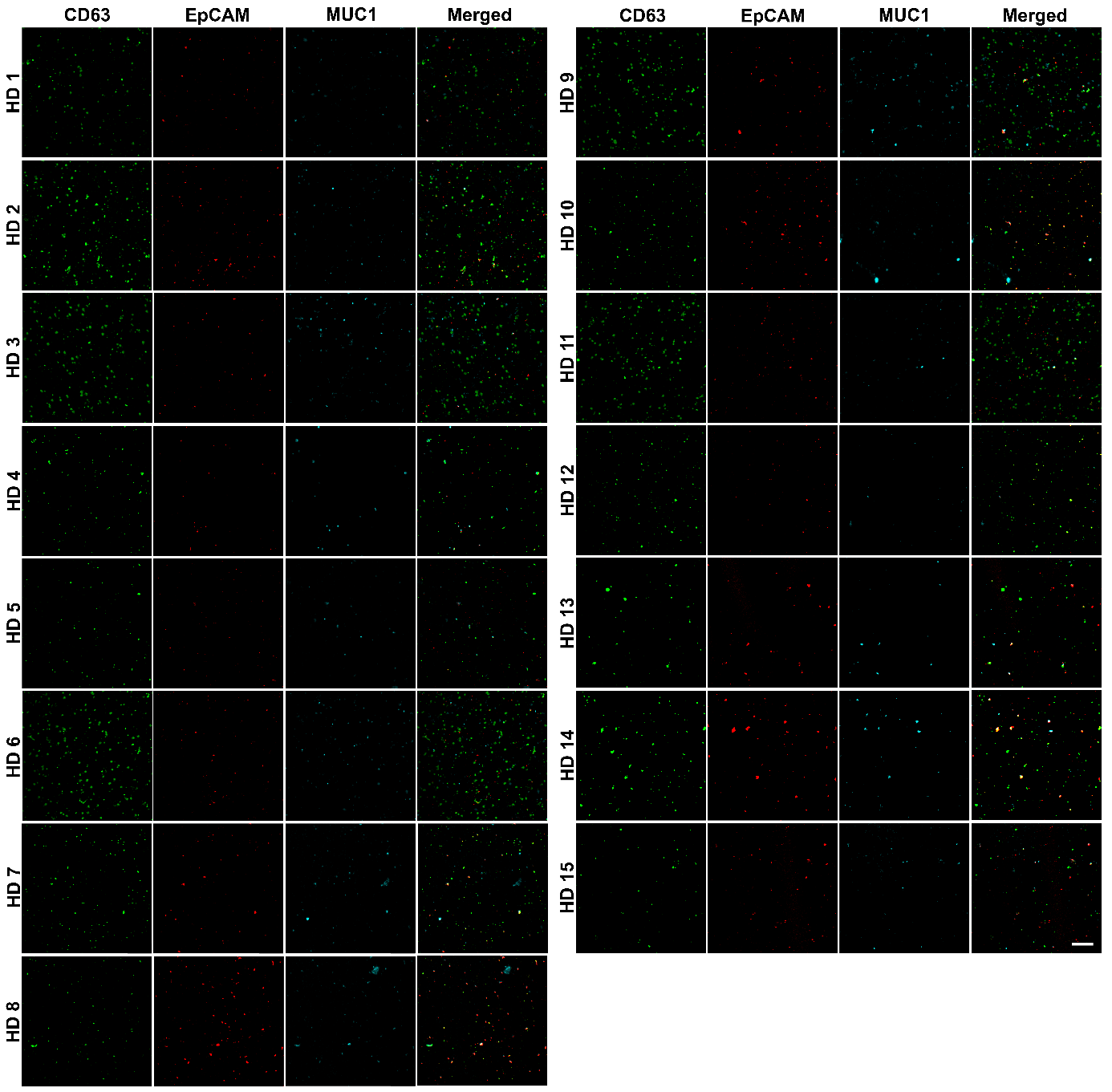


**Figure S27.** Representative fluorescent images of EV@RCA of plasma samples from healthy donors (HD, n=15). Scale bar: 10 μm.


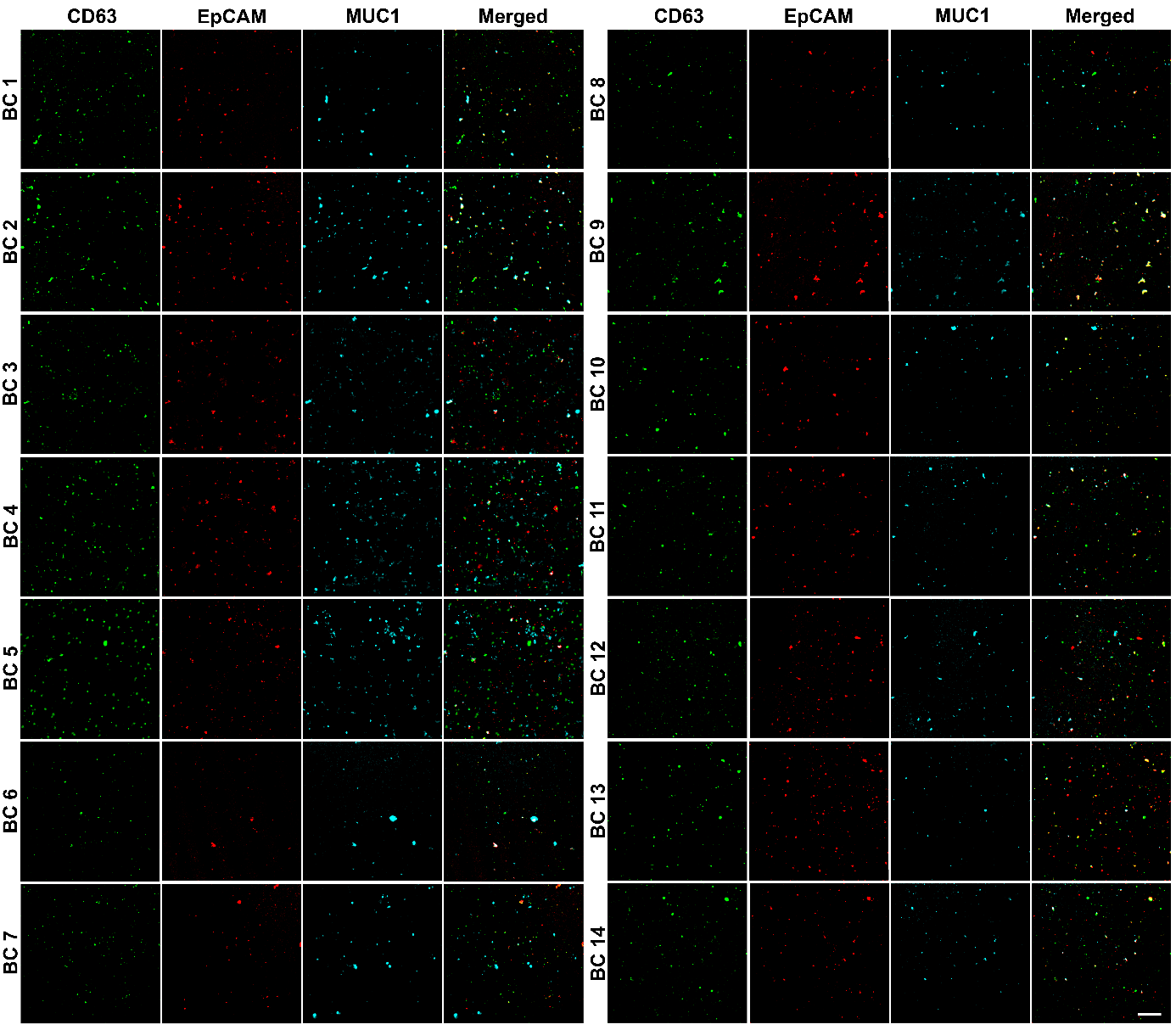


**Figure S28.** Representative fluorescent images of EV@RCA of plasma samples from breast cancer (BC, n=14) patients. Scale bar: 10 μm.


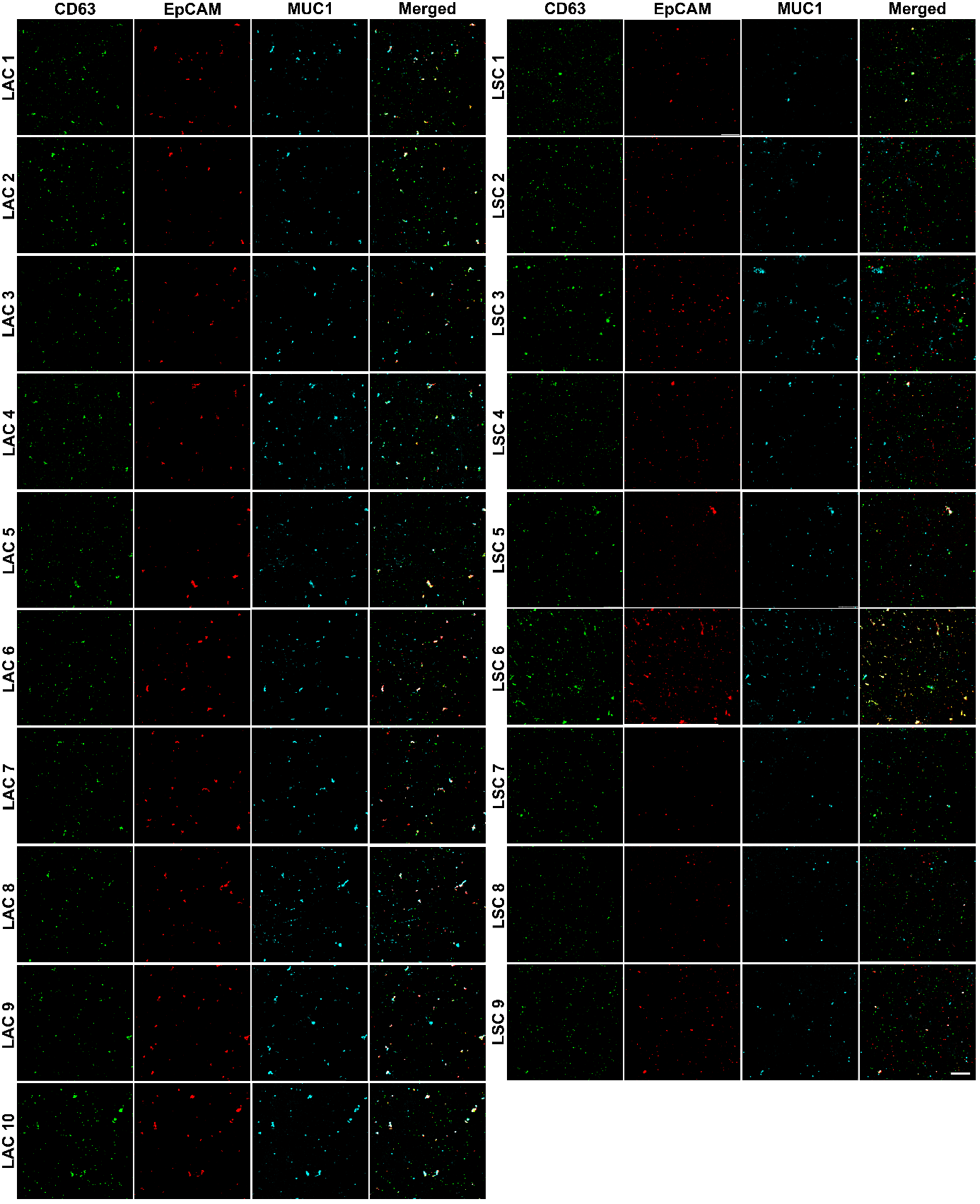


**Figure S29.** Representative fluorescent images of EV@RCA of plasma sample from lung adenocarcinoma (LAC, n=10) and lung squamous carcinoma (LSC, n=9). Scale bar: 10 μm.


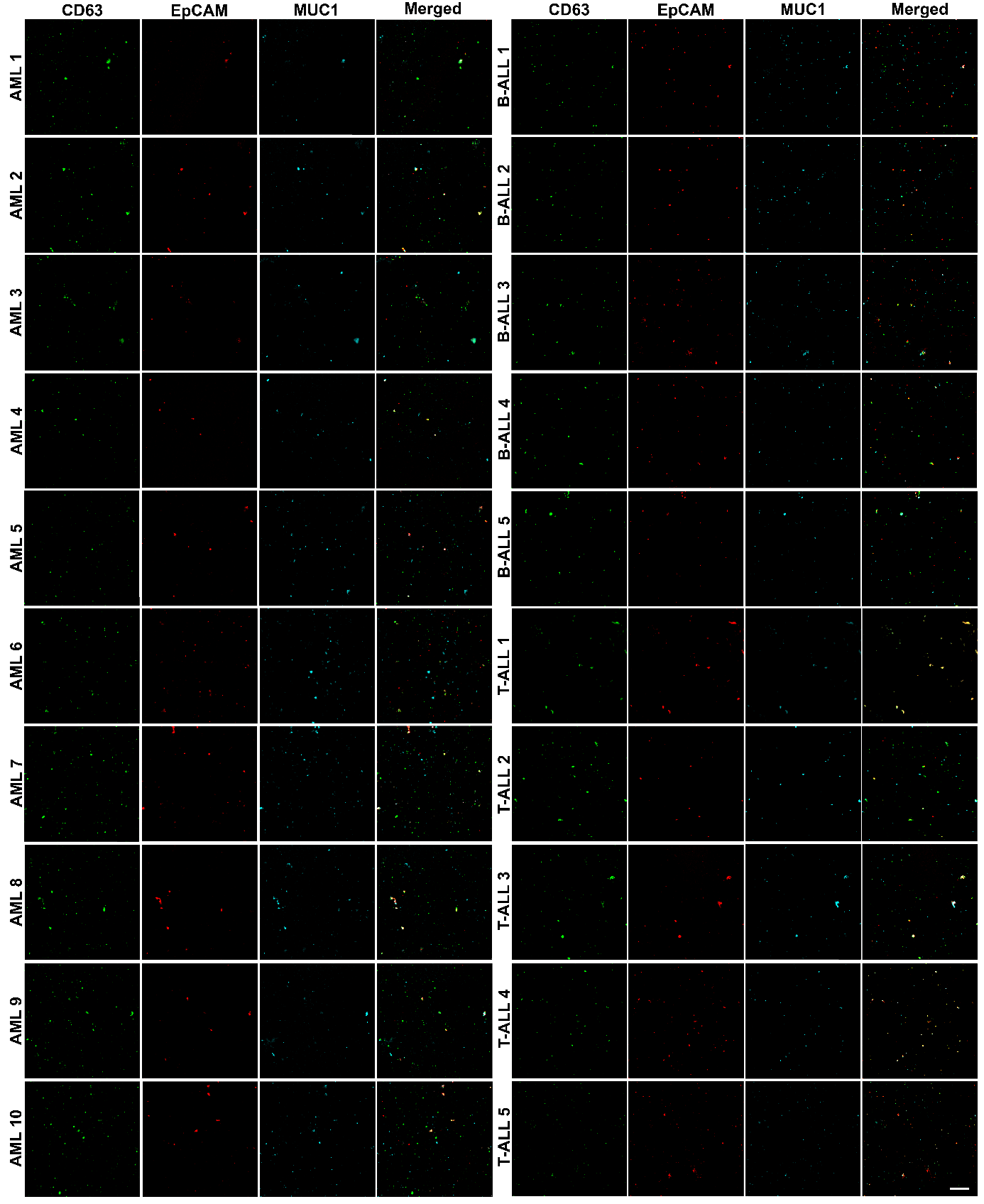


**Figure S30.** Representative fluorescent images of EV@RCA of plasma sample from acute myeloid leukemia (AML, n=10), B-cell acute lymphoblastic leukemia (B-ALL, n=5) and T-cell acute lymphoblastic leukemia (T-ALL, n=5), respectively. Scale bar: 10 μm.


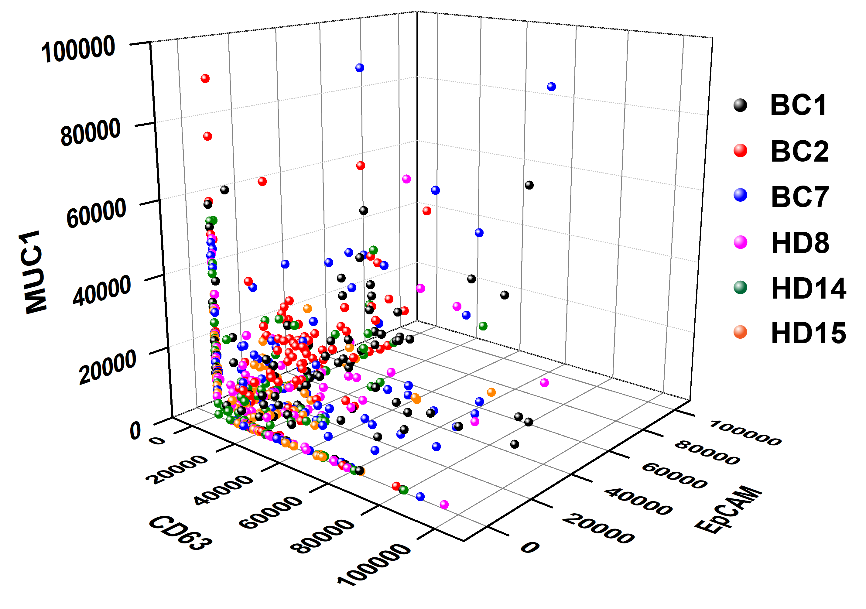


**Figure S31.** 3D scatter plots of individual-EV information from six randomly clinical samples. The fluorescence intensity of CD63, EpCAM and MUC1 were used as XYZ axis in a 3D place.


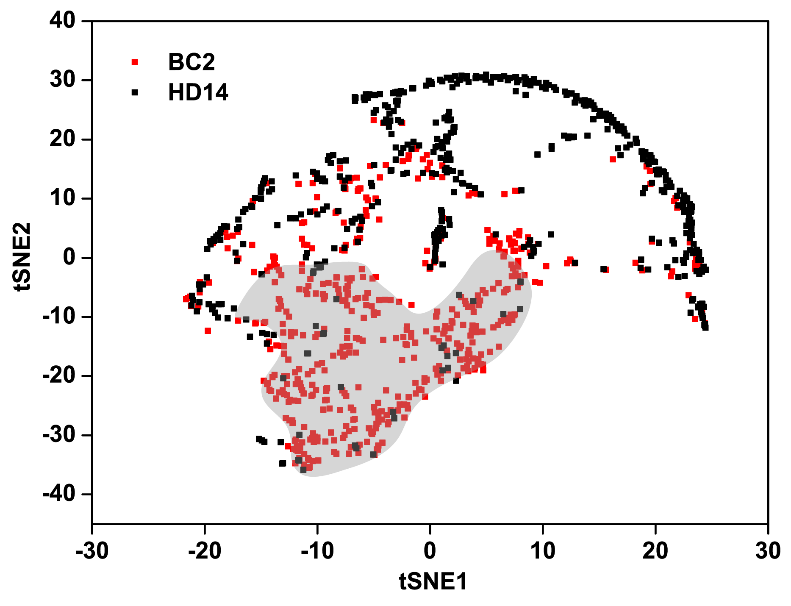


**Figure S32.** t-SNE analysis of 500 individual EV in HD14 and BC2 samples.


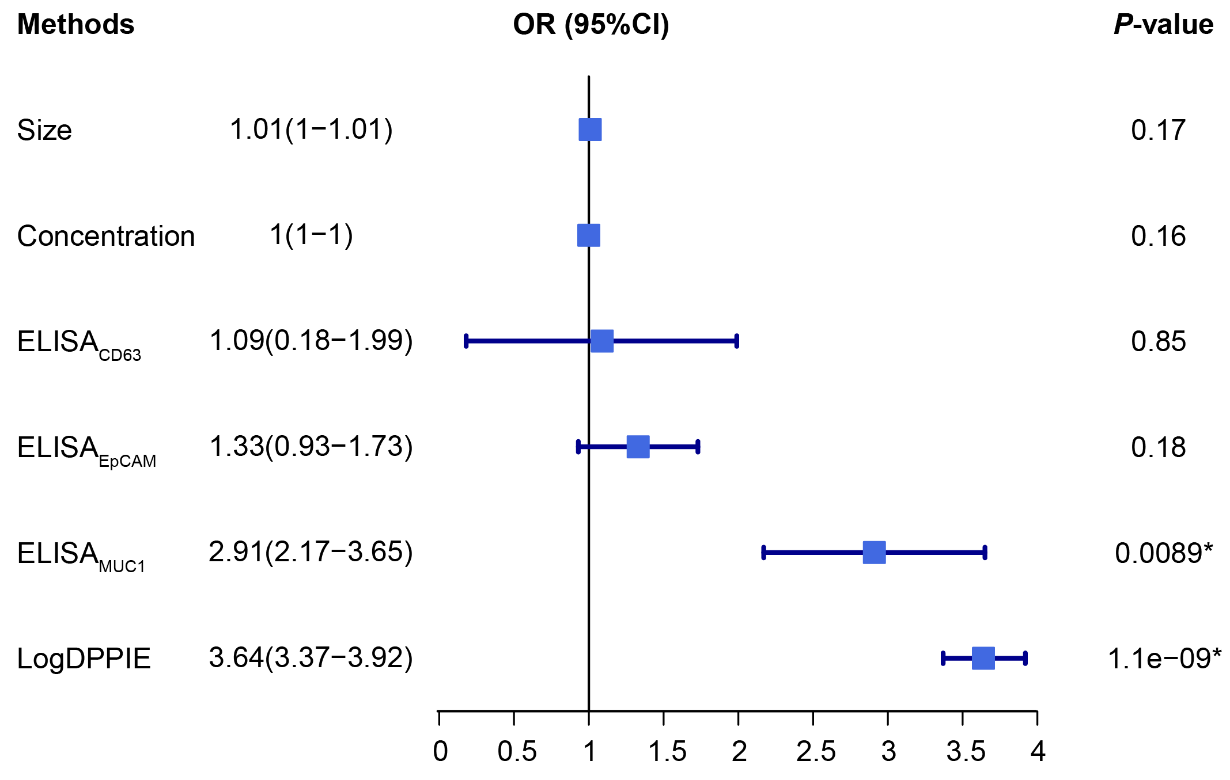


**Figure S33.** Univariate logistic regression model was used to evaluate the methods of DPPIE assay, size, concentration and ELISA measurement. OR: Odds Ratio, CI: Confidence Interval.

**Table S1.** Algorithm: Simple version of t-Distributed Stochastic Neighbor Embedding (*Journal of Machine Learning Research, 2008, 9, 2579-2605*).

| **Data**: data set $\mathcal{X=}\left\{ x_{1},x_{2},\ldots,x_{n} \right\}$, |
| --- |
| cost function parameters: perplexity *Perp*, |
| optimization parameters: number of iterations $T$, learning rate$\eta$, momentum $\alpha\left( t \right)$. |
| **Result:** low-dimensional data representation $y^{\left( T \right)}=\left\{ y_{1},y_{2},\ldots,y_{n} \right\}$. |
| **begin** |
| compute pairwise affinities $p_{j\vert i}$ with perplexity $Prep$(using $p_{j\vert i}=\frac{exp\left( {-\left\Vert x_{i}-x_{j} \right\Vert}^{2}/2\sigma_{i}^{2} \right)}{\sum_{k\neq i} exp\left( {-\left\Vert x_{i}-x_{k} \right\Vert}^{2}/2\sigma_{i}^{2} \right)}$) |
| set $p_{ij}=\frac{p_{j\vert i}+p_{i\vert j}}{2n}$ |
| sample initial solution $y^{\left( 0 \right)}=\left\{ y_{1},y_{2,\ldots,}y_{n} \right\}$ from $\mathcal{N}\left( 0,{10}^{-4}I \right)$ |
| **for** $t=1 \mathbf{to} T \mathbf{do}$ |
| compute low-dimensional affinities $q_{ij}$（using $q_{ij}=\frac{\left( 1+\left\Vert y_{i}-y_{j} \right\Vert^{2} \right)^{-1}}{\sum_{k\neq l} \left( 1+\left\Vert y_{k}-y_{l} \right\Vert^{2} \right)^{-1}}$） |
| compute gradient $\frac{\delta C}{\delta y}$（using $\frac{\delta C}{\delta y_{i}}=4\sum_{j} \left( p_{ij}-q_{ij} \right)\left( y_{i}-y_{j} \right)\left( 1+\left\Vert y_{i}-y_{j} \right\Vert^{2} \right)^{-1}$） |
| set $y^{\left( t \right)}=y^{\left( t-1 \right)}+\eta\frac{\delta C}{\delta y}+\alpha\left( t \right)\left( y^{\left( t-1 \right)}-y^{\left( t-2 \right)} \right)$ |
| **end** |
| **end** |

**Table S2.** Size distribution of EV detected by NTA in scatter (total EV) and fluorescence (CD9^+^ EV) modes.

| Mode | X10 | X50 | X90 | Mean |
| --- | --- | --- | --- | --- |
| Scatter mode (Total EV) | 57.1 | 134.7 | 238.2 | 152.9 |
| Fluorescence mode (CD9^+^ EV) | 56.3 | 138.9 | 222.4 | 143.2 |

**Table S3.** Results of size and concentration distribution of EV (dilution 200-fold with PBS) from MCF-7, Hs578Bst, B16F10 and RAW264.7 cell lines.

| **Sample** | **Size (nm)**  **Mean ± SD** | **Concentration (particles/mL)**  **Mean ± SD** |
| --- | --- | --- |
| MCF-7 EV | 157.1 ± 12.0 | 2.68×10^8^ ± 6.53×10^6^ |
| Hs578Bst EV | 137.9 ± 5.5 | 1.67×10^8^ ± 1.41×10^7^ |
| B16F10 EV | 103.7 ± 1.9 | 6.14×10^8^ ± 2.81×10^7^ |
| RAW264.7 EV | 153.9 ± 8.1 | 1.80×10^8^ ± 1.30×10^7^ |

**Table S4.** Results of size and concentration distribution of particles in plasmas (dilution 1000-fold with PBS) from healthy donors (HD, n=15).

| **Sample Number** | **Size (nm)**  **Mean ± SD** | **Concentration (particles/mL)**  **Mean ± SD** |
| --- | --- | --- |
| HD 1 | 174.7 ± 8.9 | 7.66×10^7^ ± 1.59×10^7^ |
| HD 2 | 144.0 ± 21.3 | 1.48×10^8^ ± 2.96×10^7^ |
| HD 3 | 162.7 ± 9.5 | 1.82×10^8^ ± 7.73×10^7^ |
| HD 4 | 151.9 ± 32.6 | 1.17×10^8^ ± 1.47×10^7^ |
| HD 5 | 152.5 ± 4.7 | 1.50×10^8^ ± 7.37×10^6^ |
| HD 6 | 129.8 ± 20.6 | 1.22×10^8^ ± 1.49×10^7^ |
| HD 7 | 113.2 ± 4.6 | 9.10×10^7^ ± 1.41×10^7^ |
| HD 8 | 119.7 ± 2.8 | 2.98×10^8^ ± 3.86×10^7^ |
| HD 9 | 101.5 ± 6.4 | 2.03×10^8^ ± 1.68×10^7^ |
| HD 10 | 119.6 ± 1.5 | 1.37×10^8^ ± 2.23×10^7^ |
| HD 11 | 129.1 ± 0.6 | 1.57×10^8^ ± 2.71×10^7^ |
| HD 12 | 137.6 ± 1.0 | 1.25×10^8^ ± 1.21×10^7^ |
| HD 13 | 154.6 ± 30.2 | 3.31×10^7^ ± 4.26×10^6^ |
| HD 14 | 116.0 ± 4.9 | 9.65×10^7^ ± 2.86×10^6^ |
| HD 15 | 143.7 ± 14.2 | 6.81×10^7^ ± 1.05×10^7^ |

**Table S5.** Results of size and concentration distribution of particles in plasmas (dilution 1000-fold with PBS) from breast cancer patients (BC, n=14).

| **Sample Number** | **Size (nm)**  **Mean ± SD** | **Concentration (particles/mL)**  **Mean ± SD** |
| --- | --- | --- |
| BC 1 | 111.0 ± 5.9 | 1.54×10^8^ ± 7.78×10^6^ |
| BC 2 | 158.3 ± 17.8 | 1.78×10^8^ ± 2.77×10^7^ |
| BC 3 | 128.1 ± 5.8 | 1.52×10^8^ ± 3.26×10^7^ |
| BC 4 | 173.4 ± 22.2 | 5.45×10^7^ ± 1.36×10^7^ |
| BC 5 | 153.6 ± 11.2 | 7.45×10^7^ ± 1.68×10^7^ |
| BC 6 | 151.0 ± 5.4 | 3.90×10^7^ ± 9.58×10^6^ |
| BC 7 | 137.6 ± 7.7 | 1.21×10^8^ ± 2.79×10^7^ |
| BC 8 | 161.2 ± 14.9 | 9.52×10^7^ ± 2.51×10^7^ |
| BC 9 | 129.4 ± 17.5 | 9.77×10^7^ ± 2.26×10^7^ |
| BC 10 | 106.5 ± 16.2 | 1.05×10^8^ ± 1.84×10^7^ |
| BC 11 | 148.6 ± 7.1 | 1.42×10^8^ ± 2.26×10^7^ |
| BC 12 | 176.6 ± 41.2 | 9.18×10^7^ ± 1.48×10^7^ |
| BC 13 | 166.0 ± 20.3 | 1.11×10^8^ ± 2.93×10^7^ |
| BC 14 | 170.5 ± 19.6 | 4.16×10^7^ ± 9.19×10^5^ |

**Table S6.** Results of size and concentration distribution of particles in plasmas (dilution 1000-fold with PBS) from lung adenocarcinoma (LAC, n=10) and lung squamous carcinoma (LSC, n=9) patients.

| **Sample Number** | **Size (nm)**  **Mean ± SD** | **Concentration (particles/mL)**  **Mean ± SD** |
| --- | --- | --- |
| LAC 1 | 186.8 ± 15.8 | 1.61×10^8^ ± 1.38×10^7^ |
| LAC 2 | 182.2 ± 48.0 | 8.41×10^7^ ± 1.31×10^7^ |
| LAC 3 | 157.5 ± 24.1 | 8.25×10^7^ ± 2.01×10^7^ |
| LAC 4 | 164.7 ± 31.7 | 5.61×10^7^ ± 5.11×10^6^ |
| LAC 5 | 148.3 ± 21.6 | 1.25×10^8^ ± 1.11×10^6^ |
| LAC 6 | 202.7 ± 19.9 | 2.64×10^7^ ± 4.82×10^6^ |
| LAC 7 | 147.8 ± 11.5 | 4.50×10^7^ ± 7.09×10^6^ |
| LAC 8 | 113.1 ± 20.9 | 6.93×10^7^ ± 3.04×10^7^ |
| LAC 9 | 234.4 ± 62.9 | 3.62×10^7^ ± 7.06×10^6^ |
| LAC 10 | 170.9 ± 22.2 | 4.83×10^7^ ± 1.73×10^6^ |
| LSC 1 | 148.9 ± 13.6 | 2.99×10^7^ ± 1.03×10^7^ |
| LSC 2 | 131.9 ±14.4 | 3.11×10^7^ ± 9.85×10^6^ |
| LSC 3 | 110.9 ± 7.1 | 6.55×10^7^ ± 6.10×10^6^ |
| LSC 4 | 118.5 ± 4.8 | 9.83×10^7^ ± 1.64×10^7^ |
| LSC 5 | 190.2 ± 4.4 | 8.62×10^7^ ± 1.61×10^7^ |
| LSC 6 | 252.6 ± 35.1 | 5.12×10^7^ ± 1.19×10^7^ |
| LSC 7 | 182.5 ± 14.5 | 3.80×10^7^ ± 1.42×10^7^ |
| LSC 8 | 176.6 ± 28.3 | 9.59×10^7^ ± 3.64×10^6^ |
| LSC 9 | 178.8 ± 4.3 | 1.20×10^8^ ± 2.56×10^6^ |

**Table S7.** Results of size and concentration distribution of particles in plasmas (dilution 1000-fold with PBS) from acute myeloid leukemia (AML, n=10), B-cell acute lymphoblastic leukemia (B-ALL, n=5) and T-cell acute lymphoblastic leukemia (T-ALL, n=5).

| **Sample Number** | **Size (nm)**  **Mean ± SD** | **Concentration (particles/mL)**  **Mean ± SD** |
| --- | --- | --- |
| AML 1 | 176.9 ± 19.4 | 8.21×10^7^ ± 6.82×10^6^ |
| AML 2 | 208.4 ± 21.7 | 7.49×10^7^ ± 6.92×10^6^ |
| AML 3 | 232.2 ± 13.6 | 8.72×10^7^ ± 2.16×10^7^ |
| AML 4 | 205.1 ± 31.5 | 8.03×10^7^ ± 3.12×10^7^ |
| AML 5 | 121.2 ± 33.0 | 5.56×10^7^ ± 4.56×10^6^ |
| AML 6 | 228.1 ± 31.4 | 4.24×10^7^ ± 4.08×10^6^ |
| AML 7 | 167.8 ± 35.7 | 5.37×10^7^ ± 1.15×10^7^ |
| AML 8 | 166.6 ± 19.9 | 4.97×10^7^ ± 6.90×10^6^ |
| AML 9 | 148.7 ± 18.3 | 1.90×10^8^ ± 4.53×10^7^ |
| AML 10 | 288.7 ± 10.2 | 2.18×10^7^ ± 1.06×10^7^ |
| B-ALL 1 | 160.6 ± 11.8 | 8.19×10^7^ ± 5.53×10^7^ |
| B-ALL 2 | 132.3 ± 13.7 | 7.68×10^7^ ± 3.23×10^7^ |
| B-ALL 3 | 160.0 ± 10.9 | 1.07×10^8^ ± 2.61×10^7^ |
| B-ALL 4 | 163.0 ± 17.0 | 8.19×10^7^ ± 2.10×10^7^ |
| B-ALL 5 | 153.4 ± 10.4 | 7.62×10^7^ ± 1.25×10^7^ |
| T-ALL 1 | 135.5 ± 13.0 | 4.75×10^7^ ± 4.46×10^6^ |
| T-ALL 2 | 144.3 ± 24.1 | 5.86×10^7^ ± 1.89×10^7^ |
| T-ALL 3 | 140.2 ± 7.8 | 4.01×10^7^ ± 1.07×10^7^ |
| T-ALL 4 | 195.9 ± 19.9 | 1.09×10^8^ ± 2.18×10^7^ |
| T-ALL 5 | 192.4 ± 6.0 | 7.89×10^7^ ± 1.48×10^7^ |

**Table S8.** Oligonucleotide sequences used in this work.

| **Name** | **Sequence(5’-3’)** | **Description** |
| --- | --- | --- |
| ligation template_CD63_ | **CAC CCC ACC TCG CTC CCG TGA CAC TAA TGC TA**T TTT TCG TAT CTG TGC TCA GTA AGA | The bold sequences recognition protein site-CD63 |
| ligation template_EpCAM_ | **CAC TAC AGA GGT TGC GTC TGT CCC ACG TTG TCA TGG GGG GTT GGC CTG T**TT TTT CGC ATA CTA GCT TCT GGA C | The bold sequences recognition protein site-EpCAM |
| ligation template_MUC1_ | **GCA GTT GAT CCT TTG GAT ACC CTG G**TT TTT TAG TGT CAC CAT TCC GAA CG | The bold sequences recognition protein site-MUC1 |
| padlock probe_CD63_ | P-CAC AGA TAC GTT ATA CCC GGT CGC TTT AGC CTT CTT ACT GAG | P=phosphorylation |
| padlock probe_EpCAM_ | P-TAG TAT GCG ATG TCA TTA CGA TCA TAA GTT AGG TCC AGA AGC |  |
| padlock probe _MUC1_ | P-GGT GAC ACT AGC GTA ATC GTC TCA AAT AAG GTC GTT CGG AAT |  |
| detection probe _CD63_ | Alexa 488-TTA TAC CCG GTC GCT TTA GCC T | Fluorophore-labelled detection probes for visualizing RCA amplicons |
| detection probe_EpCAM_ | Cy 5-TGT CAT TAC GAT CAT AAG TTA G |  |
| detection probe_MUC1_ | ATTO 425-GCG TAA TCG TCT CAA ATA AGG T |  |

**Table S9.** Summary of the clinical plasma samples.

| Characteristic | Gender (male/female) | Age (Mean ± SD) |
| --- | --- | --- |
| HD (n=15) | 6/9 | 40 ± 13 |
| BC (n=14) | 0/14 | 53 ± 11 |
| LSC (n=9) | 9/0 | 63 ± 7 |
| LAC (n=10) | 4/6 | 55 ± 9 |
| AML (n=10) | 0/10 | 38 ± 16 |
| T-ALL (n=5) | 3/2 | 20 ± 4 |
| B-ALL (n=5) | 4/1 | 33 ± 16 |

**Table S10.** Information of plasma sample from cancer patients.

| Types | [Sequence](javascript:;)  number | Gender | Age | Histology | Metastasis | Stage |
| --- | --- | --- | --- | --- | --- | --- |
| BC | 1 | F | 60 | Invasive carcinoma | - |  |
|  | 2 | F | 46 | Invasive carcinoma | - |  |
|  | 3 | F | 79 | invasive carcinoma | - |  |
|  | 4 | F | 48 | Breast carcinoma | - |  |
|  | 5 | F | 55 | Breast carcinoma | + |  |
|  | 6 | F | 68 | Breast carcinoma | - | III |
|  | 7 | F | 55 | Breast carcinoma | + | IV |
|  | 8 | F | 51 | Breast carcinoma | - | III |
|  | 9 | F | 45 | Breast carcinoma | + | I |
|  | 10 | F | 48 | Breast carcinoma | - |  |
|  | 11 | F | 62 | Invasive carcinoma | - | III |
|  | 12 | F | 42 | Breast carcinoma | - | IV |
|  | 13 | F | 46 | Breast carcinoma | - |  |
|  | 14 | F | 40 | Fibroadenoma | - |  |
| LAC | 1 | M | 53 | Lung adenocarcinoma | - | IV |
|  | 2 | F | 38 | Lung adenocarcinoma | - | IV |
|  | 3 | F | 63 | Lung adenocarcinoma | - | IV |
|  | 4 | F | 64 | Lung adenocarcinoma | - | IV |
|  | 5 | F | 45 | Lung adenocarcinoma | - | I |
|  | 6 | M | 62 | Lung adenocarcinoma | - | IV |
|  | 7 | M | 48 | Lung adenocarcinoma | - | IV |
|  | 8 | F | 53 | Adenocarcinoma of the left lower lobe | - |  |
|  | 9 | F | 63 | Lung adenocarcinoma | - | I |
|  | 10 | M | 65 | Poorly differentiated adenocarcinoma of the lung | - | I |
| LSC | 1 | M | 65 | Lung squamous carcinoma | - | IV |
|  | 2 | M | 60 | Lung squamous carcinoma | - | IV |
|  | 3 | M | 55 | Lung squamous carcinoma | - | IV |
|  | 4 | M | 74 | Lung squamous carcinoma | - |  |
|  | 5 | M | 70 | Lung squamous carcinoma | - |  |
|  | 6 | M | 66 | Lung squamous carcinoma | - | III |
|  | 7 | M | 67 | Lung squamous carcinoma | + | IV |
|  | 8 | M | 54 | Lung squamous carcinoma | - | II |
|  | 9 | M | 58 | Lung squamous carcinoma | - | III |
| AML | 1 | F | 52 | Acute myeloid leukemia | - |  |
|  | 2 | F | 18 | Acute myeloid leukemia | - |  |
|  | 3 | F | 44 | Acute myeloid leukemia | - |  |
|  | 4 | F | 48 | Acute myeloid leukemia | - |  |
|  | 5 | F | 21 | Acute myeloid leukemia | - |  |
|  | 6 | F | 63 | Acute myeloid leukemia | - |  |
|  | 7 | F | 39 | Acute myeloid leukemia | - |  |
|  | 8 | F | 16 | Acute myeloid leukemia | - |  |
|  | 9 | F | 37 | Acute myeloid leukemia | - |  |
|  | 10 | F | 44 | Acute myeloid leukemia | - |  |
| B-ALL | 1 | M | 17 | B-cell acute lymphocytic leukemia |  |  |
|  | 2 | F | 47 | B-cell acute lymphocytic leukemia |  |  |
|  | 3 | M | 54 | B-cell acute lymphocytic leukemia (MLL^+^) |  |  |
|  | 4 | M | 22 | B-cell acute lymphocytic leukemia（BCR/ABL^+^） |  |  |
|  | 5 | M | 25 | B-cell acute lymphocytic leukemia（BCR/ABL^+^） |  |  |
| T-ALL | 1 | M | 19 | Acute T lymphoblastic leukemia |  | IV |
|  | 2 | F | 25 | Acute T lymphoblastic leukemia |  |  |
|  | 3 | M | 25 | Acute T lymphoblastic leukemia |  |  |
|  | 4 | F | 15 | T lymphoblastic leukemia |  | III |
|  | 5 | M | 18 | T lymphoblastic leukemia |  | II |

**Table S11.** Information of plasma sample from healthy donors.

| [Sequence](javascript:;) [number](javascript:;) | Gender | Age | Histology |
| --- | --- | --- | --- |
| 1 | F | 40 | No history of cancer |
| 2 | M | 25 | No history of cancer |
| 3 | F | 54 | No history of cancer |
| 4 | M | 45 | No history of cancer |
| 5 | M | 48 | No history of cancer |
| 6 | F | 49 | No history of cancer |
| 7 | F | 23 | No history of cancer |
| 8 | M | 36 | No history of cancer |
| 9 | F | 54 | No history of cancer |
| 10 | M | 69 | No history of cancer |
| 11 | F | 25 | No history of cancer |
| 12 | M | 27 | No history of cancer |
| 13 | F | 36 | No history of cancer |
| 14 | F | 38 | No history of cancer |
| 15 | F | 30 | No history of cancer |
